# Pseudorabies Virus Infection Alters Neuronal Activity and Connectivity *In Vitro*

**DOI:** 10.1371/journal.ppat.1000640

**Published:** 2009-10-30

**Authors:** Kelly M. McCarthy, David W. Tank, Lynn W. Enquist

**Affiliations:** Department of Molecular Biology and Princeton Neuroscience Institute, Princeton University, Princeton, New Jersey, United States of America; University of Alabama at Birmingham, United States of America

## Abstract

Alpha-herpesviruses, including human herpes simplex virus 1 & 2, varicella zoster virus and the swine pseudorabies virus (PRV), infect the peripheral nervous system of their hosts. Symptoms of infection often include itching, numbness, or pain indicative of altered neurological function. To determine if there is an *in vitro* electrophysiological correlate to these characteristic *in vivo* symptoms, we infected cultured rat sympathetic neurons with well-characterized strains of PRV known to produce virulent or attenuated symptoms in animals. Whole-cell patch clamp recordings were made at various times after infection. By 8 hours of infection with virulent PRV, action potential (AP) firing rates increased substantially and were accompanied by hyperpolarized resting membrane potentials and spikelet-like events. Coincident with the increase in AP firing rate, adjacent neurons exhibited coupled firing events, first with AP-spikelets and later with near identical resting membrane potentials and AP firing. Small fusion pores between adjacent cell bodies formed early after infection as demonstrated by transfer of the low molecular weight dye, Lucifer Yellow. Later, larger pores formed as demonstrated by transfer of high molecular weight Texas red-dextran conjugates between infected cells. Further evidence for viral-induced fusion pores was obtained by infecting neurons with a viral mutant defective for glycoprotein B, a component of the viral membrane fusion complex. These infected neurons were essentially identical to mock infected neurons: no increased AP firing, no spikelet-like events, and no electrical or dye transfer. Infection with PRV Bartha, an attenuated circuit-tracing strain delayed, but did not eliminate the increased neuronal activity and coupling events. We suggest that formation of fusion pores between infected neurons results in electrical coupling and elevated firing rates, and that these processes may contribute to the altered neural function seen in PRV-infected animals.

## Author Summary

Alpha-herpesviruses, including human herpes simplex virus 1 and 2, varicella zoster virus and swine pseudorabies virus, infect the peripheral nervous system of their hosts. Symptoms often include itching, numbness, or pain. Understanding of the physiological basis for these characteristic sensory and motor anomalies remains limited. To provide more insight, we examined the electrical activity of infected neurons in culture. We used pseudorabies virus (PRV) because infected animals show strain dependent, and often, dramatic symptoms (violent scratching and self mutilation). We found that infected neurons exhibited increased action potential (AP) rates. Infected neurons also became electrically coupled, proceeding from small molecular weight dye sharing and coupled activity to large molecular weight dye sharing and complete electrical synchrony. Late in infection, cell bodies fused to form syncytia. When neurons were infected with a virulent strain that could not express glycoprotein B, a member of the viral membrane fusion complex, AP rates were not increased. Changes in electrical connectivity and dye transfer were not observed, and syncytia did not occur. These results suggest that infection induced elevated activity and electrical coupling result from virally induced membrane fusion and may underlie neurological symptoms observed during infection.

## Introduction

Alpha-herpesviruses infect a wide variety of cell types, but their hallmark is infection of the nervous systems of their hosts. Infections result in a variety of symptoms, some of which suggest an effect on motor and sensory neuron activity. For example, herpes simplex virus type 1 causes herpes labialis with the sensations of numbness and tingling [1]; herpes simplex virus type 2 causes genital herpes with the sensations of itching and pain [2]; and varicella-zoster virus causes chicken pox and shingles with sensations of itching, as well as intense pain [3]. Understanding how herpesvirus infection affects the function and connectivity of peripheral nervous system (PNS) neurons is central to understanding the cause of characteristic symptoms and pathogenesis.

Pseudorabies virus (PRV) is an alpha-herpesvirus with a broad host range that is the causative agent of Aujesky's disease in adult swine. All other susceptible animals (including most mammals, except higher primates) typically die following infection preceded by marked symptoms of PNS and central nervous system (CNS) dysfunction. Symptoms occur in a strain dependent manner and include severe pruritus with frantic self-mutilation (known as the “mad itch”), loss of motor coordination, and ataxia [4],[5].

Several early studies examined the effects of virulent PRV infection on the electrical function of infected neurons of non-natural hosts [6],[7],[8]. Extracellular recordings of rat superior cervical ganglia (SCG), a sympathetic ganglion of the PNS, showed that infected ganglia exhibited spontaneous discharges from pre- and post- ganglionic nerves in contrast to silent uninfected ganglia [6]. Simultaneous extracellular recording of pre- and post-ganglionic nerves together with intracellular recording of ganglionic neurons, revealed synchronous spontaneous discharges in pre- and post- ganglionic nerves [7],[8],[9],[10],[11]. These PRV-induced changes were hypothesized to arise from spontaneous neurotransmitter release and/or fusion of pre- and post-synaptic membranes [7],[12].

Because of their broad host range and reduced virulence, selected attenuated strains of PRV are used to trace chains of connected neurons in a variety of non-native hosts [4],[5],[13],[14],[15]. A commonly used tracer is PRV Bartha, an attenuated strain harboring a number of mutations including those in the genes encoding viral membrane glycoproteins gM [16] and gC [17], and in tegument protein UL21 [18]. This strain also carries a deletion removing the genes encoding for membrane proteins gE, gI and Us9, essential for anterograde spread of infection through neuronal circuits, as well as tegument protein Us2 [19],[20],[21]. Recent tracing studies employing attenuated PRV Bartha strain derivatives reported that the electrophysiological properties of infected hamster and rat CNS neurons were unaffected by infection, despite behavioral signs of neurological defects late in infection prior to death [22],[23],[24],[25].

In this study we describe the effects of infection of virulent and attenuated strains of PRV on neuronal activity and connectivity using whole-cell patch recording methods on dissociated primary cultures of embryonic rat SCG neurons. The PRV strains used included a virulent PRV Becker strain expressing GFP (PRV 151), an avirulent mutant derivative of PRV Becker (PRV 233) that cannot express gB, an essential component of the viral membrane fusion complex [26], and an attenuated PRV Bartha strain expressing GFP (PRV 152). We report that neurons infected with PRV 151 exhibited elevated rates of action potential (AP) firing and spikelet-like events that correlated with formation of small fusion pores between adjacent cell bodies. Over the course of infection, PRV 151 neurons become increasingly dye and electrically coupled, resulting in synchronous activity. By 40 hpi, multinucleated neuronal syncytia were formed. When neurons were infected with a complemented gB null mutant, PRV 233, AP firing rates were not increased above uninfected neurons. No changes in electrical connectivity or dye transfer were observed, and formation of multi-nucleated syncytia never occurred. Interestingly, neurons infected with the attenuated tracing strain PRV152 were substantially delayed in all the infection-induced changes observed for virulent infection.

We conclude that the viral membrane fusion complex (the gB, gH and gL proteins) produces fusion pores several hours after infection that enable ions to flow between neurons resulting in electrical (not synaptically mediated) coupling. At early times, the pores are small, open to diffusion of small molecular weight dyes such as Lucifer Yellow. These pores effectively couple the electrical activity of neurons. At later times, pores are larger, open to diffusion of larger molecular weight rhodamine-dextran conjugates. These larger pores completely synchronize firing events between involved neurons. The delay, but not elimination, of all firing and coupling events in PRV 152 infected neurons implies that mutations in the PRV Bartha genome modulate action or localization of the viral membrane fusion complex in neurons.

## Results

### PRV infects and replicates in dissociated SCG neurons

**Figure 1:**
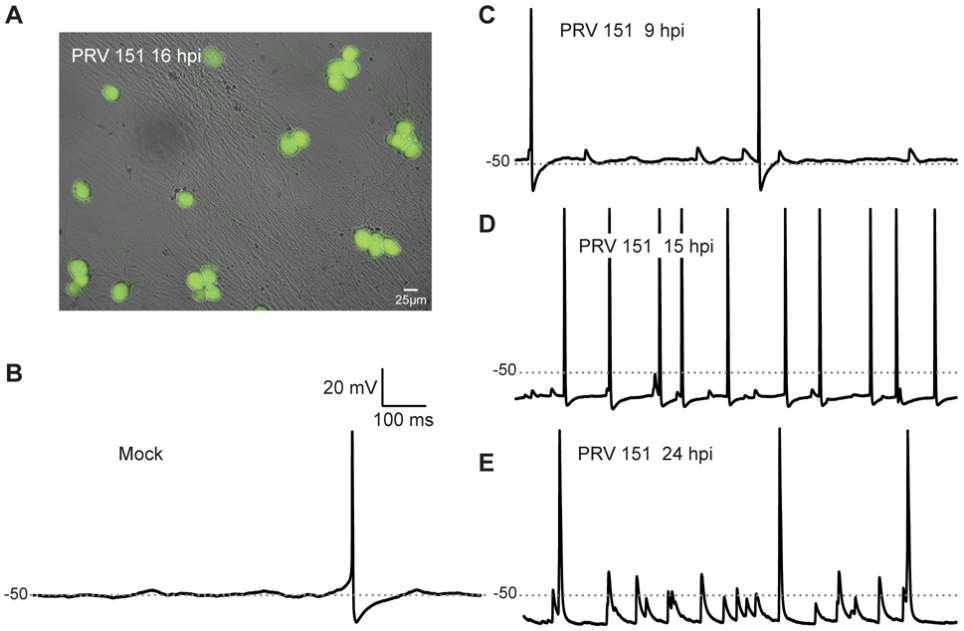
Elevated rates of electrical activity observed in PRV infected neurons. (A) PRV 151 infected neurons show uniform GFP fluorescence 15 hpi. (B–E) Typical voltage traces of mock and PRV 151 infected neurons obtained using whole-cell patch recordings in current clamp. Traces from 4 different neurons are shown. −50 mV is marked by a gray dotted line for resting membrane potential comparison.

Previous studies investigating changes in electrophysiology of PRV infected neurons used widely varying techniques, including intracellular sharp recordings of infected SCG neurons *in situ* and patch clamp recordings in brain slices of infected animals. In these studies, it was difficult to determine if and when a recorded neuron was infected. To overcome these limitations, we used cultures of dissociated rat sympathetic ganglion (SCG) neurons, free of replicating epithelial and support cells. After one week in culture, (see methods), the cell bodies of some SCG neurons had divided once or twice before terminal differentiation and were typically found either in clusters of 2–6 cells. These neurons also formed an extensive network of axons (Figure 1A). We infected cultures with sufficient virus to ensure infection of all cell bodies. In most of our studies we used PRV 151, a virulent PRV Becker recombinant that expresses diffusible GFP. Fluorescence was visible by 4 hpi in all neurons across each culture and remained strong for at least three days following infection (Figure 1A).

### PRV infection induces significant electrophysiological changes in infected neurons *in vitro*

*In situ* recordings from rat SCG tissue infected with PRV 151 revealed significant changes in electrophysiological properties, including highly elevated rates of spontaneous AP firing and synchronous activity on pre- and post- ganglionic nerves [6],[8],[9]. To determine whether similar changes would be seen in cultured PRV infected SCG neurons, we examined action potential (AP) firing rates using whole cell patch recordings in current clamp mode. In control mock-infected neurons, AP firing was rare, and slow rising and decaying excitatory post-synaptic potential (epsp)-like depolarizations were only occasionally observed on top of the resting membrane potential (Figure 1B). As PRV 151 infection progressed, neurons began to fire APs at elevated rates compared to mock-infected neurons (Figure 1C–E). Infected neurons also displayed smaller spikelet-like events and had significantly hyperpolarized resting potentials, despite increases in AP firing rates (Figure 1C–E). These results demonstrate that electrophysiological changes qualitatively similar to those observed in tissue were observed under our culture conditions.

### PRV infected neurons fire APs at elevated rates by 8 hours post infection

**Figure 2:**
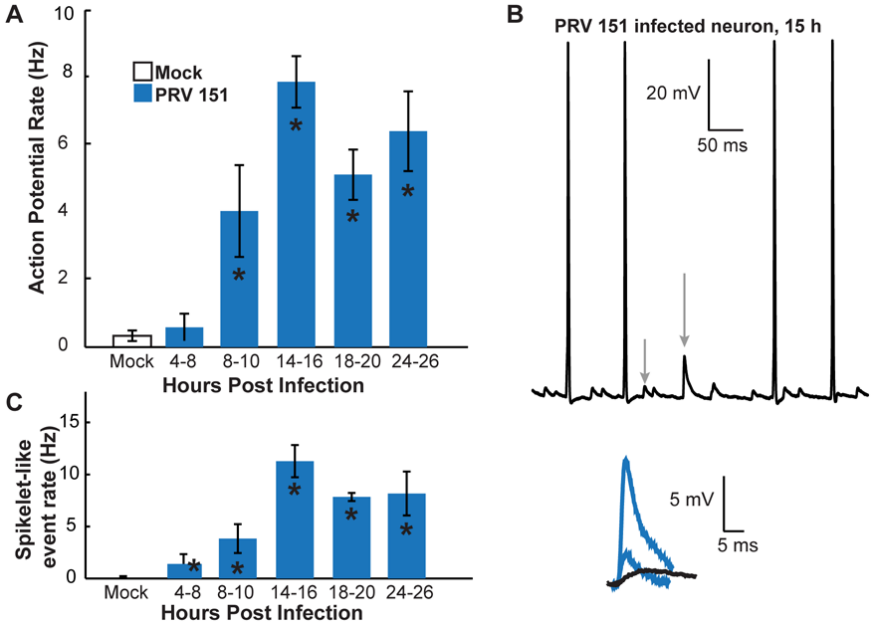
Onset of PRV-induced elevated AP and spikelet-like event rates. (A) Mean AP rates of PRV 151 infected neurons from 4 to 24 hpi. PRV 151 infected neurons fired more frequently than mock-infected neurons after 8 hpi. (B) Example whole-cell recording from a PRV 151 infected neuron at 15 hpi. Grey arrows indicate examples of spikelet-like events. These events are shown in blue, overlaid with an example of slower rising and decaying epsp in black. (C) Mean rates of spikelet-like events in PRV 151 infected neurons. PRV 151 infected neurons began to show events by 4 hpi as noted in panel A. Mock n = 51, 4–8 hpi PRV 151 n = 8, 8–10 hpi n = 8, 14–16 hpi n = 29, 18–20 hpi n = 4, and 24–26 hpi n = 14. In this and following figures asterisks (*) on bars correspond to a statistical significant difference of P<0.05 compared to the mock condition. Asterisks above brackets indicate a statistical significant difference of P<0.05 between strains. Error bars indicate standard error.

We next characterized the time course and magnitude of these changes. We first recorded from neurons at specific times following infection (4–8,8–10,14–16,18–20 and 24–46 hpi) and compared their AP firing rates with that observed under mock-infection. Mock-infected SCG neurons had a mean AP firing rate of 0.3±0.2 Hz (Figure 2A). This same low firing rate was seen in PRV 151 infected neurons between 4 and 8 hpi (Figure 2A). However, by 8 hpi PRV 151 infected neurons began to fire APs at a mean elevated rate of 4.0±1.4 Hz (Figure 2A). By 14–16 hpi the PRV 151 infected neurons were firing at rates 10–25 fold greater than uninfected neurons; most had rates greater than 5 Hz (86.2%), with a mean rate of 7.9±0.8 Hz (Figure 2A,B). Infected neurons continued to fire APs at elevated rates at 24 hpi and did so until at least 72 hpi, the longest incubation period assayed (Figure 2A, data not shown).

### PRV infected neurons show signs of electrical coupling

In addition to elevated AP firing rates, PRV infected neurons showed brief sub-threshold depolarizations similar to spikelets observed in electrically coupled neurons [27],[28] (Figure 2B, gray arrows). These putative electrical coupling events had fast rise and decay rates and were often followed by a shallow after-hyperpolarization. They were distinctly different from smaller more slowly rising and decaying, chemically mediated, excitatory postsynaptic potentials (epsps) observed in mock-infected neurons (Figure 2B, blue vs black traces) [29],[30]. We identified these events based on rate of rise (see Methods) to distinguish them from any slower rising and decaying epsps. In the following, we will refer to these events as “spikelet-like events”. Typically when spikelet-like events were observed, they occurred in several size classes, similar to previously described spikelets (Figure 2B, blue traces) [31]. Spikelet-like events began to occur at elevated rates above mock-infection by 4 hpi in PRV 151 infected neurons, and continued to occur at high rates until at least 24 hpi (Figure 2C).

### Elevated AP and spikelet-like event rates are unaffected by somatic current injections

**Figure 3 ppat-1000640-g003:**
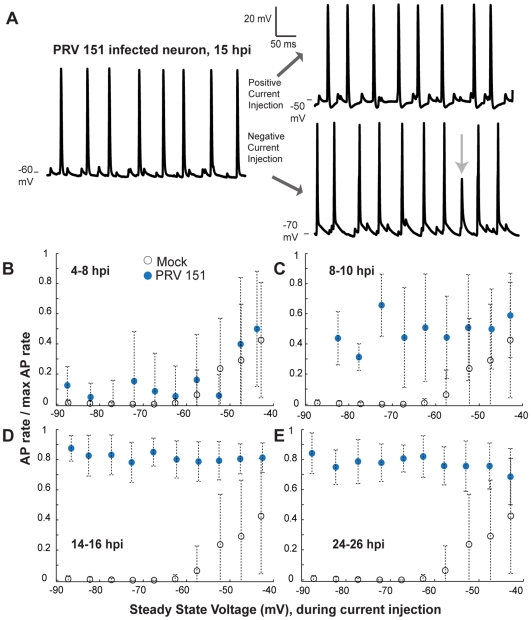
Despite large cell body current injections, elevated AP rates remain relatively unaffected. (A) Traces from a single PRV 151 infected neuron at 15 hpi. Left, voltage trace during no current injection, right, voltage trace during positive (top) and negative (bottom) current injections. Voltage levels indicated at the lower edge of each trace. (B–E) Relationship between steady state voltage and AP firing rates during current injections. The total number of APs during a given voltage bin (5 mV) was divided by the maximum AP rate for that cell (max rate during any injection −0.15 to 0.14 nA). Ratios were averaged and plotted against steady state voltage. Note relatively unchanging AP rates of infected neurons as infection progressed. Mock n = 24, 4–8 hpi PRV 151 n = 9, 8–10 hpi n = 7, 14–16 hpi n = 15, 24–26 hpi n = 7.

Current injections through the patch electrode were used to examine if PRV 151 infection affected the relationship of steady state voltage and AP firing rate of SCG neurons. In mock-infected neurons, AP firing rate could be elevated by injection of depolarizing currents (Figure 3B). A mean depolarization of 6.1±0.8mV was required to reach AP threshold (n = 22, Figure 3B). Between 4 and 8 hpi, PRV 151 infected neurons responded much like mock-infected neurons to current injections and required the same level of voltage to reach AP threshold (5.5±2.4mV, n = 5 Figure 3B).

By 8–10 hpi, PRV 151 infected neurons with elevated AP and spikelet-like event rates had a weaker response to current injections: hyperpolarizing current typically was unable to silence the neuron (Figure 3C). As a result, no threshold could be calculated for these neurons.

At 14–16 hpi, PRV 151 infected neurons showed no relationship between AP firing rate and steady state voltage achieved by current injection (Figure 3A,D). In a few cases, strong cell body hyperpolarization was able to reduce the AP amplitude. Full APs were replaced by spikelet-like events of large amplitude (Figure 3A, arrow). As a result, these neurons did exhibit a weak dependence of AP firing rate on injected current, as explained in Figure S1A–F.

By 24–26 hpi, PRV 151 neurons showed AP firing rates that were largely independent of injected current over a broad range (PRV 151 5/7, Figure 3E). As mentioned above, the replacement of full APs with large spikelet-like events during strong hyperpolarization accounts for the apparent dependence of AP rate on injected current (Figure S1D–F). The combined rate of spikelet-like events and full APs remained the same as found when no current was injected infected neurons starting at 14–16 hpi (Figure S1G–J).

The appearance of spikelet-like events and AP firing rates independent of steady state voltage during current injections strongly suggests that APs did not initiate in the cell bodies, but rather occurred in synchrony with large spikelet-like depolarizations in electrically isolated axons.

### PRV infected neurons have hyperpolarized resting membrane potentials and altered AP shapes

**Figure 4 ppat-1000640-g004:**
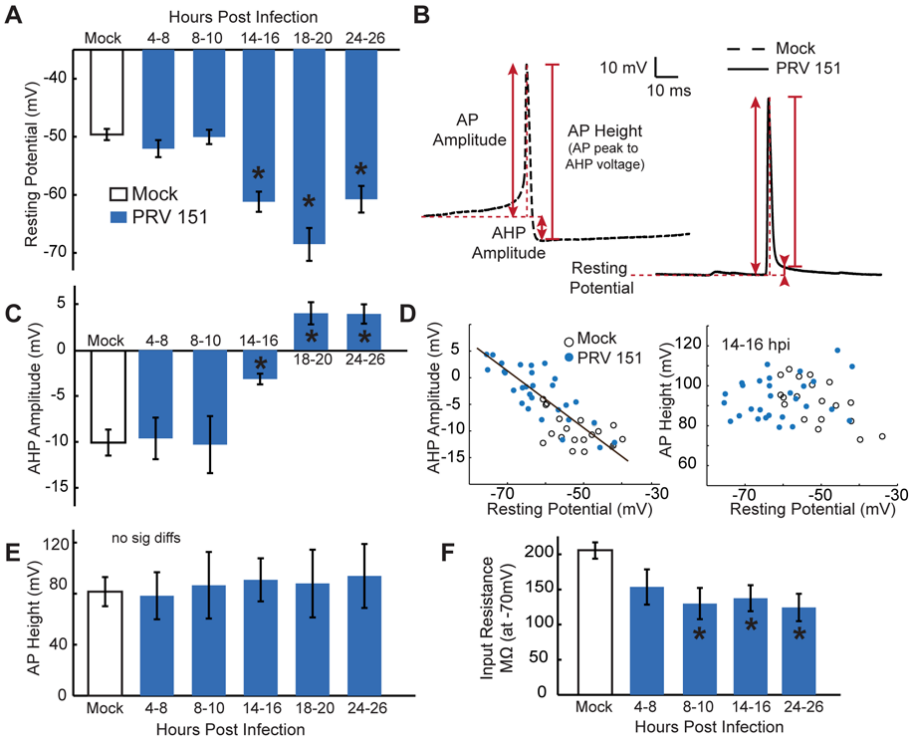
Time course of PRV induced changes in resting membrane potential and AP shape. (A) Resting membrane potentials of PRV 151 infected neurons were similar to mock-infected neurons before 14 hpi and were hyperpolarized for the duration of the times measured. (B) Example traces of spontaneous AP, representative of means at 14–16 hpi. Horizontal dotted red lines denote resting membrane potential, the first solid vertical arrowed line indicates AP amplitude, the second solid vertical arrowed line represents AHP amplitude, and the third vertical red line represents AP height (difference between the peak of AP amplitude and the voltage measured for AHP). (C) AHP amplitude remained comparable to mock-infected neurons until 14 hpi, and was more positive for the duration of the time course. (D) More positive AHP amplitudes correlated with resting voltages similarly for PRV 151 and mock-infected neurons, example shown at 14–16 hpi. Linear fits: in black, to all conditions, y = −.50x−34.3 R2 = 0.68, to mock condition y = −.20x−20.15 R2 = 0.26, and to PRV 151 at 14–16 hpi, y = −0.54x−36.17 R2 = 0.73. No trend was observed in AP height as a function of resting voltage. (E) Total peak AP to AHP minimum in infected neurons did not change significantly from mock infected for at least 24 hours. (F) Input resistance of infected neurons became increasingly lower in magnitude as infection proceeded. Resistance measured from points in a 10 mV range surrounding −70 mV. (A–E) Mock n = 19, 4–8 PRV 151 n = 12, 8–10 hpi n = 7, 14–16 hpi n = 9, 18–20 n = 4, 24–26 n = 14. (F) Mock n = 14, 4–8 hpi PRV 151 n = 5, 8–10 hpi n = 4, 14–16 hpi n = 9, 24–26 hpi n = 5.

We next determined whether PRV infection induced changes in resting membrane potential or AP shapes. Early in infection (4–10 hpi), neurons infected with PRV 151 had resting potentials and AP shapes comparable to mock-uninfected neurons. By 14–16 hpi, despite high firing frequency, resting potentials of PRV 151 infected neurons were strongly hyperpolarized compared to uninfected control neurons (−59.5±1.9mV vs −49.5±1.1mV, p<0.01). Infected neurons remained significantly hyperpolarized until at least 24 hpi (Figure 4A). Additionally, the input resistance of infected neurons was reduced compared to mock infected neurons as infection progressed (Figure 4F).

The shape of spontaneous APs also was changed by PRV 151 infection. AP shape was quantified by measuring the AP amplitude (difference in voltage between the absolute peak of AP), AHP amplitude (difference in voltage between resting membrane potential and the voltage 11 ms after AP peak) and total AP height (difference between the absolute peak of AP and the voltage 11 ms after AP peak) (Figure 4B). During the first 10 h of PRV infection, the shape of spontaneous APs remained nearly identical to that of mock-infected neurons (Figure 4B,C,E). However, by 14–16 hpi, PRV 151 infected neurons showed altered AP shapes. Infected neurons' AHPs were significantly reduced in amplitude (−3.2±5.3mV range −13.1 to 4.4mV vs −10.0±3.0mV range −13.9 to −4.0mV) after 14 hours of infection, corresponding with the hyperpolarization of membrane potentials (Figure 4C,D). Total AP height remained unchanged throughout infection up to 24 hpi (Figure 4E).

Since these changes in shape coincided with marked hyperpolarization, we explored whether hyperpolarization alone was responsible for the change in shape by injecting hyperpolarizing and depolarizing current. As seen in Figure 3A, during depolarizing current injections infected neuron APs showed larger AHP amplitudes and smaller AP amplitudes, while further hyperpolarization led to smaller AHP amplitudes and larger AP amplitudes (Figure 3A). However, the total AP height was unchanged. This also held true of neurons across all treatments during no current injection, as shown in Figure 4D. These results suggest that the differences in AP shape observed during PRV infection are an indirect result of the changes in resting membrane potential.

In addition, the AP shape at threshold of AP initiation had a generally sharper inflection in infected neurons. This change is evident by inspection of the right versus left traces in Figure 4B. Sharp inflection points at the onset of AP initiation have also been observed in cell body recordings of APs back propagated from axons [31],[32]. The observation of brief rapid deflections supports our contention that APs were initiating in the axons of infected neurons firing at elevated rates.

### PRV infection induces small and large molecular weight dye coupling, correlated membrane potentials, and late neuronal syncytia formation

The shapes and characteristics of the spikelet-like events were suggestive of electrical coupling analogous to that observed with gap junction mediated electrical synapses between neurons. We used well characterized dye transfer methods used to characterize gap junctions [33],[34] to determine if we could detect any direct diffusion pathways between two infected neurons. Both Lucifer Yellow (457 MW), the traditional low molecular weight dye used in gap junction characterization, and Texas-red conjugated dextrans of much larger molecular weight (3,000, 10,000, and 40,000 MW) were used to fill individual neurons from the patch pipette [33],[35]. In addition to dye transfer measurements, dual patch electrodes were used to record from two neurons simultaneously to analyze the temporal correlation of dye transfer, APs, and spikelet-like events in the two neurons. The effect of current injected in one cell on the membrane potential of the second cell was also evaluated to determine electrical coupling ratios.

For Lucifer Yellow (LY) dye transfer measurements, whole cell recordings using dye-filled electrodes were obtained from randomly chosen cell bodies at various time points after infection. Recordings were maintained for 10–15 minutes to allow dye to diffuse into the cell body from the electrode. The electrode was then removed and a one-hour incubation period was provided to allow time for LY to diffuse into its processes and to any connected neurons. Because LY fluorescence is not easily distinguished from GFP, we used the parent strain PRV Becker, which exhibits identical electrophysiological changes identical to those induced by PRV 151 infection (data not shown).

**Figure 5:**
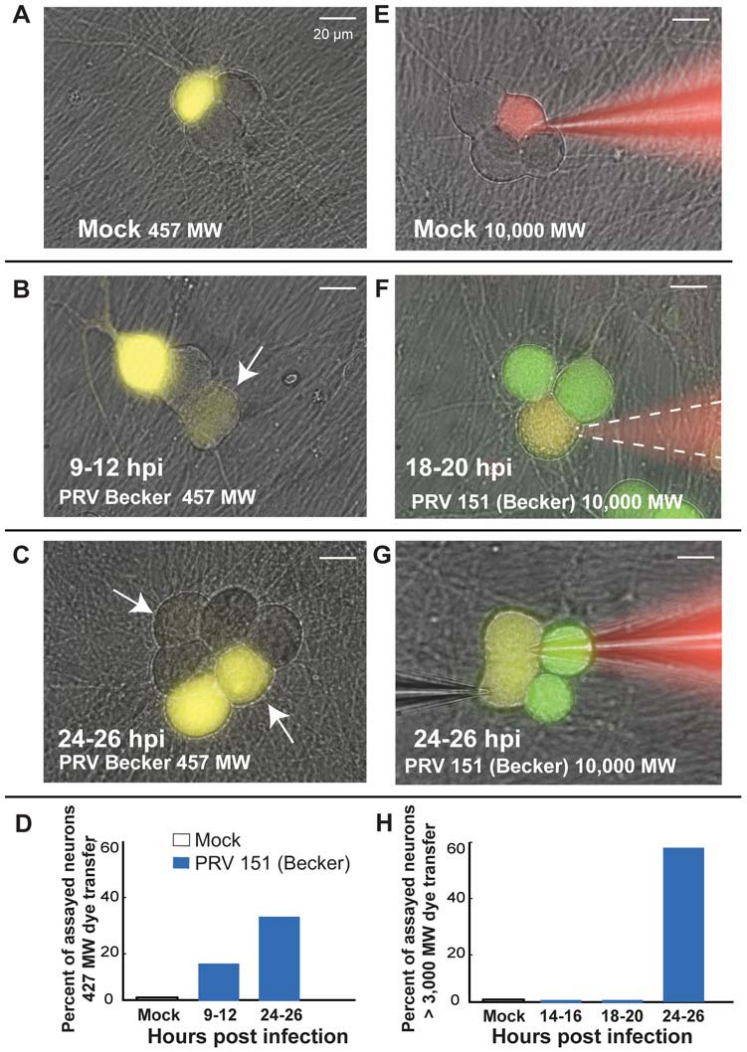
Increasingly larger molecular weight dyes transfer between neurons as infection proceeds. (A) Lucifer yellow (LY, 457 MW) dye did not transfer from filled mock-infected cell bodies to other cell bodies (0/10). Images represent overlays of excited Lucifer yellow onto bright field images. (B–C) Transfer of LY to other cell bodies could be seen in PRV 151 infected neurons by 9 hpi,(2/12). White arrows indicate cell bodies that contain dye transferred from the filled neuron. Transfer of LY from filled cell bodies to other cell bodies was more common in PRV 151 infected neurons by 24 hpi (2/6). Note that PRV 151 infected cell bodies that contained transferred dye were generally not adjacent to filled cell body, and were exclusively to non-adjacent cell bodies early (9–12 hpi). (D) Percentage of neurons assayed in which dye spread from the filled cell body to another cell body. (E) Texas-red dextran conjugates (3,000–40,000 MW) to not transfer from filled cell bodies of mock-infected neurons to other cell bodies (0/9). (F–H) Large dye transfer between infected neurons occurs late in infection (14–16 hpi, 0/9, 18–20 hpi, 0/9, 24–26 hpi, 11/19). Neurons selected for dye filling were chosen as potentially fused cells based on outer membrane contours by light microscopy. Green – GFP expressed from viral genome, Red – Texas-red dextran conjugate, Yellow – overlap of green and red images. Panel F, bright field image was recorded following fluorescence image and pipette removal; pipette location is indicated by a dashed while line.

When mock-infected cell bodies were filled with LY containing electrodes, cell bodies and axonal processes (>20um, range 1–4 2.3±0.3) were filled with dye, but no other cell bodies or processes were filled (Figure 5A). Similarly, neurons infected for less than 8 hours with PRV Becker retained LY within their cell bodies and prominent processes with no evidence of transfer into other cells. However, by 9 hpi, randomly filled PRV Becker infected neurons began to share LY with other non-adjacent cell bodies (Figure 5B,D). At this time the mean number of processes labeled with dye was similar to that of mock infected neurons (range 1–15, 4.5±1.1). By 24–26 hpi, many PRV Becker infected neurons shared LY with adjacent and distant cell bodies (Figure 5C,D). In addition to dye continuity with other cell bodies, LY filled infected neurons also had a larger number of contiguous labeled processes by 24–26 hpi (range 2–19, 7.0±2.5, p<0.05). Some of these processes are likely primary axons from distant cell bodies. Dye may have transferred from the filled neurons to the axons passing over the cell body. In general, these LY transfer results demonstrate that PRV infection produced a gradual development of small pores between infected neurons that enabled flow of small molecular weight dyes.

By measuring transfer of large molecular weight dyes (Texas-red dextran conjugates) known to be too large to pass between gap junctions (>2,000 MW), we observed that larger pores formed later in PRV 151 infection. Cell bodies were filled with various Texas-red dextran conjugates by diffusion from a patch electrode (3,000–40,000 MW) for 10–15 minutes prior to imaging. Because Texas-red fluorescence was easily distinguished from that of GFP, PRV 151 was used in these experiments.

In both mock-infected and early PRV infected (<24hrs) neurons, no transfer of the Texas red conjugates was obvious (Figure 5E–F,H). However, by 24 hpi, conjugates of at least 40,000 MW were readily transferred from filled PRV 151 infected neurons to adjacent infected neurons (Figure 5G,H, Table 1).

**Table 1:** Large MW dye transfer between PRV 151 infected SCG neurons.

	PRV 151			
Size (MW)	3000	10000	40000	total
**14–16 hpi**	0/5	0/4	–	**0/9**
**18–20 hpi**	0/4	0/4	0/1	**0/9**
**24–26 hpi**	5/10	3/6	3/3	**11/19**
**Mock**	0/4	0/5	–	**0/9**

Individual cell bodies were filled with Texas-red dextran conjugates and assayed for transfer into adjacent cell bodies at either 18–20 or 24–26 hpi.

**Figure 6:**
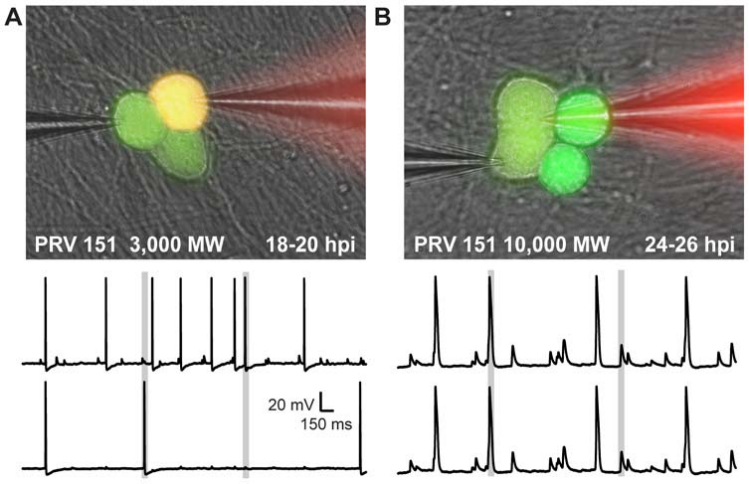
Large MW dye transfer between infected neurons corresponds with near complete electrical synchrony. Dual whole cell recordings from pairs of adjacent neurons that did or did not exhibit large MW dye transfer (≥3,000 MW) at 18–20 hpi and 24–26 hpi were used to examine the electrical continuity of adjacent infected cell bodies. (A) An example pair showing neither dye transfer, nor complete electrical continuity. Time aligned traces shown are representative of those recorded. (B) An example pair showing both large MW dye transfer and complete electrical coupling. Note that in the case of large MW dye transfer, pairs of neurons showed complete electrical synchrony, including spikelet-like events and AP firing (A, grey boxes); however, when large MW dye was not transferred, spikelet-like events occurred at the same time as AP firing in the adjacent neuron (B, gray boxes). Pair described in B is example from Figure 4F.

To relate dye transfer to electrophysiological activity, dual whole-cell patch recordings were performed on several pairs of infected neurons. As shown in Figure 6 during the time period of elevated firing rate, recorded pairs that did not share large molecular weight Texas red- dextrans had correlated events in the two recordings. Fast depolarizing potentials exhibited by one neuron corresponded to full AP firing of the adjacent, non-filled neuron, and vice versa nearly 100% of the time (3/3, Figure 6A). In these cases, DC current that was injected into one cell produced a small change in membrane potential in the second cell (Figure S2A). The coupling ratios, a measure of the change in membrane potential in the second cell divided by that produced in the current-injected cell were >0.1. However, when large molecular weight Texas red dextrans transferred between adjacent cell bodies, the membrane voltage traces of the two recorded neurons were identical and had a coupling ratio close to 1 (Figure 6B, Figure S2B). From an electrical standpoint, the membranes of the two cell bodies were contiguous.

**Figure 7:**
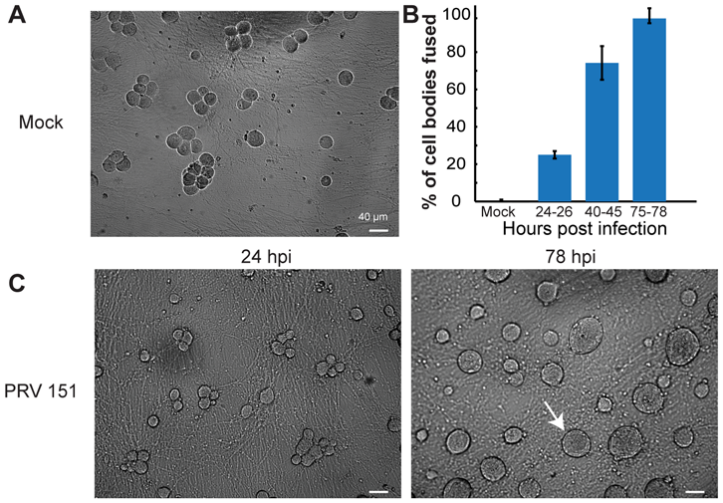
PRV infection leads to fusion of adjacent cell bodies into multi-nucleated syncytia. (A,C) Representative 10× fields of view of PRV 151 and mock-infected neurons at 24 and 78 hpi. White arrows indicate examples of neuronal fusion. (B) Quantification of the percentage of cell bodies assayed for fusion, as determined by angle between outer membrane appositions or the area of large multi-nucleated syncytia (see Methods, Figure S3). Mock n = 4, PRV 151 24–26 hpi n = 4, 40–45 hpi n = 3, 75–78 hpi, n = 2. For each >85 individual cell bodies were assayed from random fields of view.

At late times after infection (>18 hrs) signs of membrane fusion were visible by bright field microscopy. Specifically, membrane contours of the junction between adjacent cell bodies of some infected neurons relaxed. A distinct vertex is normally observed where the membranes of two adjacent cell bodies come into contact; however, as infection progressed, this vertex became increasingly difficult to resolve. This change suggested that cell bodies were fusing. To quantify the extent of fusion across a population of neurons in a culture dish, we examined the relationship between dye transfer and the angle at the vertex formed by the outer contours of adjacent neuronal cell bodies that either shared or did not share large MW dye. As shown in Figure S3A, outer angles greater than 125 degrees between pairs unambiguously corresponded to non-dye sharing pairs, while those with larger angles indicated large MW dye sharing (examples, Figure 7C, arrow and Figure S3B). We therefore used vertex angle measurements from bright field images of a population of neurons in randomly chosen fields of view as an unbiased indirect estimate (compared to selecting pairs for dual electrode recording) of fusion. As infection progressed, there was an increasing fraction of fused cells (Figure 7B,C). These data suggest clusters of infected neurons transitioned from non-dye coupled, to small MW dye coupled, to large MW dye sharing and as infection proceeded. This process continued and resulted in formation of cell body syncytia by 78 hpi (Figure 7B,C).

### The viral membrane protein gB is necessary for PRV induced elevated rates of AP firing and spikelet-like events, electrical coupling and neuronal fusion

As shown above, PRV infected neurons become both electrically and dye coupled leading to the formation of cell body syncytia. Syncytia formation and cell-cell fusion are well known to occur in non-neuronal cells as a result of PRV infection. This process requires the viral fusion glycoproteins gB, gH, and gL [36]. A null mutation in any of these genes blocks the entry of free viral particles to initiate infection, as well as eliminates cell-cell spread and the formation of multi-nucleated syncytia. Therefore, we infected neurons with the gB null mutant virus 233, derived from PRV Becker, expressing GFP, to determine the role of virally induced fusion in the above observations [26]. This mutant is propagated in gB complementing cells so viral particles produced contain gB on their host derived membrane envelopes and are able to enter a cell. Once inside, viral infection proceeds normally, but no gB is expressed and despite particle production, no further spread can occur [37],[38],[39].

**Figure 8:**
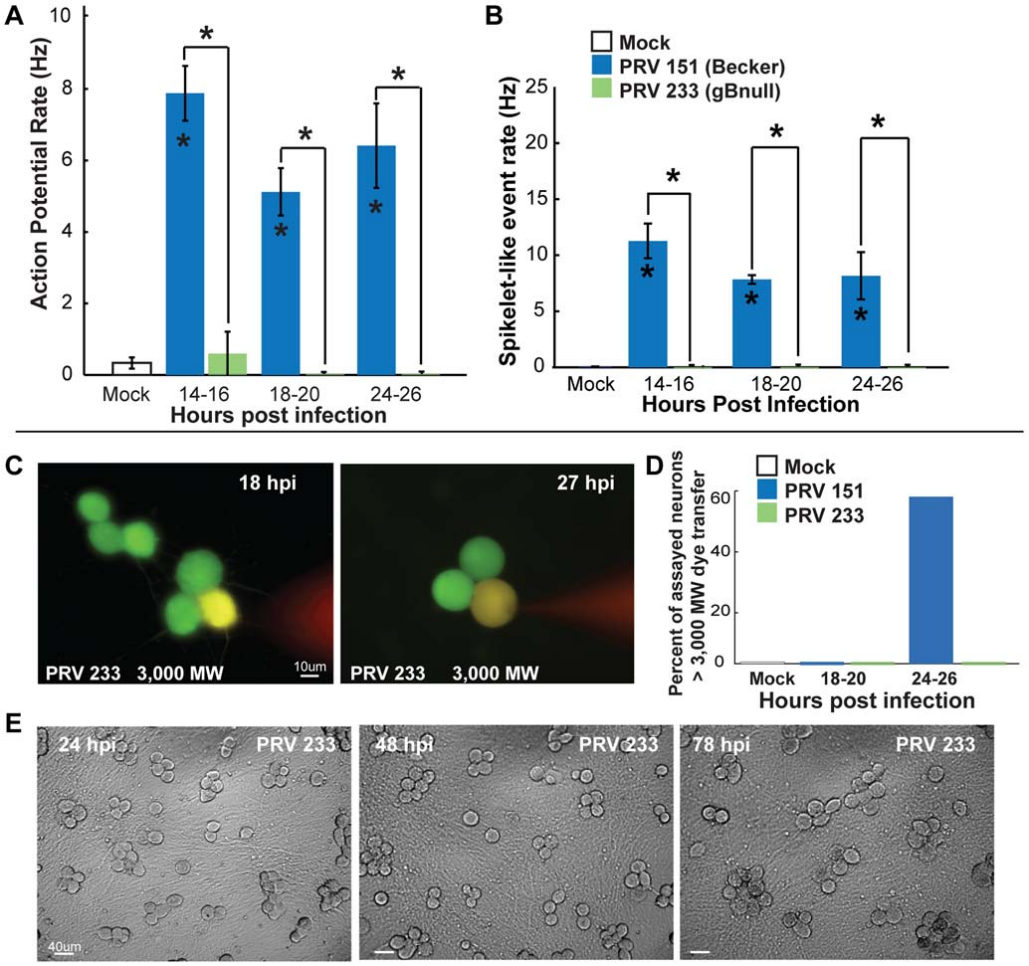
The viral membrane fusion protein gB is required for elevated rates of AP firing and fusion of infected neurons. (A–B) Mean AP firing and spikelet-like event rates of mock, PRV 151 and PRV 233 infected neurons. (PRV 233 14–16 hpi n = 16, 18–20 hpi n = 8, 24–26 hpi n = 15). (C–D) Large MW dye filling of infected gB null PRV 233 infected neurons and quantification. At both 18 and 27 hpi, dye was contained and neurons were not firing elevated rates of APs or spikelet-like events (PRV 233 18–20 hpi, 0/4, 24–26 hpi 0/4). (E) Representative fields of view of neurons infected with PRV 233 gB null mutant. Note no visible fusion of cell bodies over the course of infection. Mock and PRV 151 data replotted from Figures 2–5.

Strikingly, gB null infected neurons showed no evidence of elevated rates of AP firing or spikelet-like events as late as 24 hpi, the last time point assayed (Figure 8A,B). These infected neurons had normal resting potentials until 20 hpi, but by 24 hpi resting potentials were mildly hyperpolarized compared to mock-infected neurons (−54.0±1.2mV vs −49.6±1.0mV P<0.05). However, gB null PRV infected neurons were significantly less hyperpolarized than PRV 151 infected neurons at 24 hpi (−60.8±2.3mV and −62.0±2.2mV P<0.05). The input resistance of gB null PRV infected neurons also was slightly reduced by 24 hpi, but was not significantly different from mock-infected neurons (137±28 vs 206±12MΩ, slope at −70mV n = 4,14). Like the AP firing rate for mock infected neurons, AP firing rates of PRV 233 infected neurons had a positive relationship to steady state voltage (or injected current) (data not shown).

Individual gB null PRV infected neurons showed no signs of pore formation or fusion events. Infected neurons filled with dextran conjugated Texas red (≥3000 MW) showed no dye transfer to cell bodies with closely opposing membranes (0/4 at 18–20 hpi and 0/4 at 24–26 hpi, Figure 8C,D). These infected neurons also showed no cell body-cell body fusion by bright field microscopy, as late as 78 hpi, the latest time point assayed (Figure 8E).

The gB null mutant results strongly suggest that virally mediated membrane fusion events produce pores that allow ions and dyes to flow between cell bodies. These pores increase in size over time and their appearance correlates with the induction of rapid AP firing and spikelet like events. gB null infected neurons are not hyperpolarized at early times after infection, but do exhibit modest hyperpolarization at late times.

### Strain dependent onset of PRV induced elevated AP firing rates

The attenuated PRV Bartha strain and its derivatives (e.g., PRV 152) have been widely used to identify synaptically connected neurons. PRV Bartha derivatives spread only from post-synaptic to pre-synaptic neurons (retrograde spread only) and all infected animals have markedly reduced symptoms compared to virulent strains. The PRV Bartha genome carries several characterized mutations that affect anterograde spread of infection between neurons, for efficient spread of infection in the retrograde direction, and for virulence [4]. It the context of this report, it is important to stress that PRV Bartha has no known mutations in the gB coding sequences and is expresses gB at equivalent levels [40].

Given that previous reports had indicated that in animals infected with PRV 152, neurons showed no signs of abnormal electrophysiology [24], we used our *in vitro* system to compare PRV 151 and PRV 152 infected neurons. SCG neurons infected with PRV 152 produced titers of infectious virus comparable to those infected with PRV 151 and the time course of infection (as measured by GFP expression) was comparable (data not shown).

**Figure 9:**
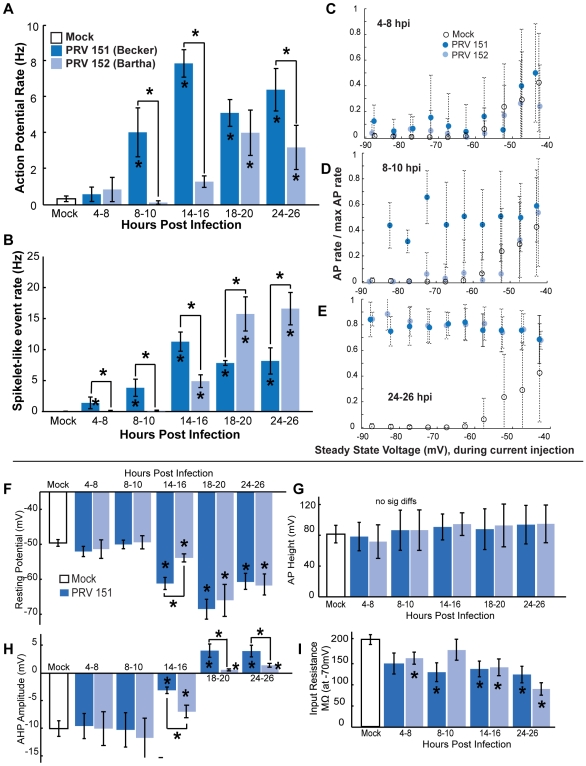
Delayed onset of PRV induced changes by attenuated PRV 152. (A,B) Onset of increased mean AP firing rate of PRV 152 infected neurons was delayed until 18 hpi and onset of spikelet-like events was delayed until 14 hpi. (C–E) Relationship between steady state voltage and AP firing rates during current injections, plotted as described in Figure 2. Note delay to unchanging AP rate as a function of steady state voltage until 24–26 hpi. (F) Resting membrane potentials of PRV 152 infected neurons were similar to mock-infected neurons before 14 hpi and were hyperpolarized for the duration of the times measured. (G) AHP amplitude remained comparable to mock-infected neurons until 14 hpi, (H) Total peak AP to AHP minimum in PRV 152 infected neurons did not change significantly from mock-infected for at least 24 hours. (I) Input resistance of PRV 152 infected neurons became increasingly lower in magnitude as infection proceeded. (A–I) PRV 152 data re-plotted with PRV 151 data from Figure 2. (A–B) PRV 152 4–8 hpi n = 9, 8–10 hpi n = 8, 14–16 hpi n = 41, 18–20 hpi n = 6, and 24–26 hpi n = 15. (C–E) PRV 152 4–8 hpi n = 7, 8–10 hpi n = 7, 24–26 hpi n = 8. (F–H) 4–8 hpi PRV 152 n = 4, 8–10 hpi n = 7,3, 14–16 hpi n = 6, 18–20 n = 6, 24–26 n = 11. (I) PRV 152 4–8 hpi n = 5, 8–10 hpi n = 7, 14–16 hpi n = 9, 24–26 hpi n = 5.

Unlike PRV 151 infections, PRV 152 infected neurons did not show elevated rates of AP firing by 8 or 16 hours post infection (Figure 9A). However, 10 hours later (18 hpi) PRV 152 infected neurons showed elevated AP firing rates, indistinguishable from PRV 151 infected neurons (Figure 9A). Similarly, PRV 152 induced AP firing rates, like those induced by PRV 151, continued unabated until at least 72 hpi.

The onset of PRV 152 spikelet-like events was also delayed by 10 hours, beginning at 14–16 hpi. By 18 hpi 66.7% of PRV 152 infected neurons showed spikelet-like events at rates greater than 10 Hz (vs. 0% PRV 151 infected), and 86.7% by 24 hpi (vs. 35.7% PRV 151). These rates were higher than those of PRV 151 infected neurons at comparable time points. However, the elevated mean AP firing rates were lower during periods in which PRV 152 infected neurons showed higher rates spikelet-like events compared to PRV 151 infected neurons. Despite this difference, the total event rate, including APs and spikelet-like events, in PRV 152 infected was not significantly different compared to PRV 151 infected neurons at 18 or 24 hpi (Figure 9A,B).

AP firing rates independent of steady state voltage during current injection was also delayed by 10 hours in those infected with PRV 152 (Figure 9C–E). At 4–8 and 8–10 depolarization required to reach AP threshold was not altered compared to mock infected neurons (4.1±1.9mV, 5.3±2.4mV vs 6.0±0.8mV, n = 5,5,22). At 14–16 hpi, PRV 152 infected neurons started to show a similar trend as PRV 151 infected neurons 8–10 hpi, namely that hyperpolarizing currents were typically unable to completely silence the neurons (6/19). By 24–26 hpi the majority of PRV 152 infected neurons showed AP firing rates with no dependence on steady state voltage during current injection (5/8, Figure 9E). A weak relationship between absolute injected current level and AP firing rate after increased AP firing rates began, explained in (Figure S4A–F). As seen in PRV 151 infected neurons, the combined AP and spikelet-like event rate could not be controlled current injection.

PRV 152 infected neurons also showed changes in resting potential and AP shape similar to PRV 151 infected neurons. By 14–16 hpi PRV 152 infected neurons became significantly hyperpolarized compared to mock-infected neurons (−52.5±1.0mV, p<0.05, Figure 9F), showed a similar change in AHP amplitude (−7.5±1.1mV range −15.9 to 7.0mV, Figure 9H) and showed a sharp infection in rise to AP at threshold. Changes in AP shape corresponded with hyperpolarization of resting membrane potential. Input resistance of PRV 152 infected neurons also was significantly reduced after infection (Figure 9I).

**Figure 10:**
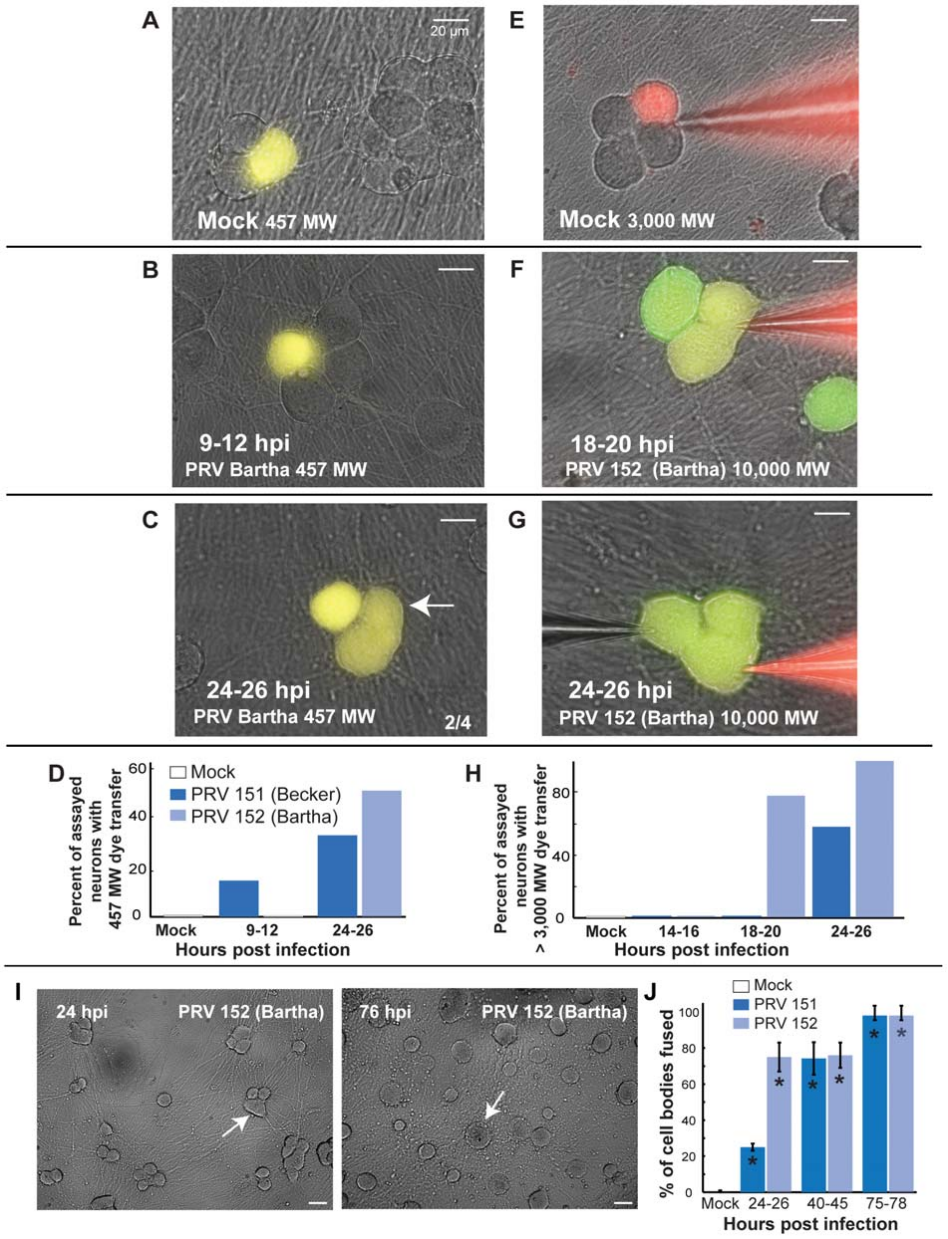
PRV 152 infected neurons follow a similar progression of fluorescent dye transfer and fusion of cell bodies. (A–D) Lucifer yellow (457 MW) dye transfer between PRV 152 infected neurons was delayed until 18 hpi (9–12 hpi, 0/8, 24–26 hpi (2/4). (E–H) Large MW (≥3,000 MW) dye transfer occurred by 18–20 hpi between PRV 152 infected neurons, earlier than occurred between PRV 152 infected neurons (14–16 hpi, 0/3, 18–20 hpi, 7/9, 24–26 hpi, 9/9). (I–J) Representative fields of view of PRV 152 infected neurons and quantification. Note earlier cell body-cell body fusion between PRV 152 infected neurons compared to PRV 151 infected neurons, consistent with that observed in large MW dye experiments. (I–J) PRV 152 24–26 hpi n = 4, 40–45 hpi n = 2 and 75–78 hpi, n = 2. Data were collected displayed as described in Figures 5 and 7.

Membrane fusion events occurred in PRV 152 infected neurons, but were significantly delayed. Neurons infected with PRV Bartha (the PRV 152 parent) showed no evidence of LY transfer at 9–12 hpi (0/8, Figure 10A). By 18 hpi, both LY and Texas red conjugates transferred between infected neurons (Figure 10B–H, Table 2). While transfer of large MW fluorescent dye was observed earlier between PRV 152 infected neurons than those infected with PRV 151 (18 vs 24 hpi), transfer was only observed between adjacent and not distant cell bodies (Figure 10B–C,F–G). Similarly to those infected with PRV 151, by 24 hpi PRV 152 infected neurons showed LY dye transfer to a larger number of adjacent processes (9–12 hpi, range 2–4, 2.9±0.6, 24–26 hpi range 2–19, 8.0±2.7). And, when Texas red conjugates were shared, neurons showed complete electrical synchrony (3/3 vs 0/2 not sharing). We also noted that in bright field microscopy, PRV 152 infected neurons showed more cell body-cell body fusion at 24 hpi as compared to PRV 151 infected neurons, consistent with earlier adjacent cell body dye transfer (Figure 10J).

**Table 2:** Large MW dye transfer between PRV 152 infected SCG neurons.

	PRV 151				PRV 152			
Size (MW)	3000	10000	40000	total	3000	10000	40000	total
**14–16 hpi**	0/5	0/4	–	**0/9**	0/3	–	–	**0/3**
**18–20 hpi**	0/4	0/4	0/1	**0/9**	3/5	3/3	1/1	**7/9**
**24–26 hpi**	5/10	3/6	3/3	**11/19**	3/3	4/4	2/2	**9/9**
**Mock**	0/4	0/5	–	**0/9**				

Individual cell bodies were filled with Texas-red dextran conjugates and assayed for transfer into adjacent cell bodies at either 18–20 or 24–26 hpi. PRV 151 and mock data re-displayed from Table 1.

These experiments indicate that infection with PRV Bartha delayed, but did not eliminate increased neuronal activity and coupling. Since there is no published evidence that PRV Bartha has mutations in gB or in membrane fusion activity, these results indicate that other mutations in the PRV Bartha genome modulate action or localization of the viral membrane fusion complex in neurons.

## Discussion

### PRV infection of sympathetic neurons *in vitro* results in changes in neuronal activity and connectivity

Previous studies of rodents infected with PRV indicated that PNS ganglia, particularly the SCG, exhibited altered activity characterized by elevated AP firing rates. We used the same PNS neurons in primary culture to demonstrate that PRV infection strongly affects electrophysiology and connectivity. Moreover, using an avirulent (PRV 233) and an attenuated PRV strain (PRV 152), we were able to identify the primary and modulating events that lead to this altered activity. We found that both PRV 151 and PRV 152 infected neurons began to fire APs at elevated rates, but firing induced by PRV 152 was significantly delayed (at least 10 hours delayed). The shape of the induced APs for both infections was unusual and the induced spikelet-like events were clearly different from epsps characteristic of synaptic events. We found that the induced AP and spikelet-like events correlated with formation of small fusion pores between adjacent cells. These pores were revealed by dye transfer experiments and by observation of electrical coupling of fused neurons. Over the course of infection, the pores became larger as demonstrated by passage of large molecular weight Texas red dextran conjugates. When neurons were infected with the avirulent gB mutant, PRV 233, AP firing rates did not change and were indistinguishable from those seen in uninfected neurons. No changes in electrical connectivity or dye transfer were observed, and formation of multi-nucleated syncytia never occurred for as long as three days post infection.

These results with the gB null mutant are consistent with the hypothesis that the primary initiator of altered firing of PRV infected neurons is the viral membrane fusion complex (consisting of the gB, gH and gL proteins). This complex produces fusion pores several hours after infection that enable ions to flow between neurons resulting in electrical (synaptically mediated) coupling. At early times, the pores are small, open to diffusion of small molecular weight dyes such as Lucifer Yellow. These pores effectively couple the electrical activity of neurons. At later times, the pores are larger, open to diffusion of larger molecular weight rhodamine-dextran conjugates. These larger pores completely synchronize firing events between involved neurons. The delay, but not elimination, of all firing and coupling events in PRV 152 infected neurons implies that mutations in the PRV Bartha genome modulate action or localization of the viral membrane fusion complex in neurons. It is conceivable that the gB protein plays roles outside of the fusion complex that affect firing. Tests of the idea that the fusion complex is indeed the major initiator of firing would entail studies of gH and gL null viruses, which should behave like gB null mutants in these studies.

### Elevated AP firing rates despite hyperpolarized membrane voltage

Resting membrane potentials of PRV 151 and PRV 152 infected neurons were significantly hyperpolarized. This finding is surprising as hyperpolarization is usually associated with a decrease in firing rate. Hyperpolarization may be a homeostatic response of infected neurons to significantly increased rates of depolarizing inputs [31],[32],[41]. This hypothesis suggests that when PRV induced AP and spikelet-like events begin, infected neurons compensate by shifting the balance of currents responsible for maintenance of membrane potential. We did observe a small, yet significant, hyperpolarization of neurons infected with a gB null strain, which did not exhibit increased firing event rates. These data suggest hyperpolarization also may reflect a more general response to PRV infection. Infection alters transcription of a number of genes, including increased transcript levels of cyclooxygenase-2 [42],[43]. Increased expression of this gene has been shown to lead to membrane hyperpolarization in a variety of neurons [44],[45],[46]. Further work is necessary to determine the mechanism of PRV induced hyperpolarization.

### Electrical coupling and fusion events involving infected neurons

PRV 151 infected neurons show evidence of electrical coupling early by 8 hours after infection that correlate with increased AP firing and spikelet-like events. Neither AP firing or spikelet-like events could be silenced by hyperpolarizing cell body current injections and the shape of APs that recorded from cell bodies was characteristic of that of back-propagated APs, both suggesting events originated at outside, electrically coupled sites. Infected neurons showed progressively stronger electrical coupling and transfer of larger dye molecules as infection continued. Dual recordings revealed reciprocal connections early, in which APs recorded from one neuron occurred at the same time as a smaller, dampened depolarization (or spikelet-like event) occurred in the other. Later, adjacent neurons showed synchronous events later in infection, in which APs and spikelet-like event timing was identical in adjacent neurons. In addition, late in infection, cell bodies fused to form multi-nucleated syncytia. These data suggest that fusion of neuronal membranes occurs first on a small scale, possibly transiently, early at sites of membrane apposition. Later, these small pores expand, leading to more cytoplasmic continuity between neurons. Finally, late in infection, these pores expand resulting large rounded neuronal syncytia. PRV 152 infected neurons exhibit identical properties except that everything is delayed by at least 10 hours. As observed for PRV 151 infected neurons, increased firing rates are correlated with appearance of dye transfer events and electrical coupling.

Previous *in vitro* studies have suggested that alpha-herpesvirus infection can provoke neuronal membrane fusion. Scanning electron microscopy of virulent PRV infected rat CNS neurons *in vitro* showed evidence of neuronal membrane fusion [47]. Spread of the fluorescent lipophilic dye DiI from PRV infected SCG axons to adjacent infected epithelial cell plasma membranes suggested fusion of plasma membranes [48],[49]. Intracellular and patch clamp recordings of cultured rat sensory neurons infected with a syncytial strain of herpes simplex virus 1 showed elevated rates of AP firing, spikelet-like events and synchronous activity, similar to those described in this study [43],[50],[51]. These effects were not observed in non-syncytial strains. It was suggested that neurons became electrically coupled by fusion of axonal processes, and that this coupling supported spontaneous electrical activity initiated by spontaneous APs. We have extended these observations to demonstrate fusion directly by dye transfer between both adjacent and distant infected neurons in conjunction with electrophysiological recordings.

### Induction of elevated rates AP firing and neuronal coupling require gB

Changes observed in AP firing rates, spikelet-like events, electrical coupling, dye transfer and formation of multi-nucleated syncytia after PRV infection were not seen in neurons infected with PRV 233, a gB null mutant. gB is an essential PRV membrane protein found in the virion envelope and on the plasma membranes of infected cells. gB is required as part of a viral protein complex for fusion of extracellular particles with host cell membranes and for spread of infection from one cell to another including trans-synaptic spread among neurons *in vivo* and *in vitro* [39],[52],[53]. Because gB mutants complete all other viral replication, assembly, and egress processes, we conclude that gB promoted fusion events are necessary for PRV induced changes in neuronal activity and connectivity. It remains to be seen if expression of viral fusion machinery (gB, gH, and gL) is alone sufficient.

Others have reported that when antiviral drugs, such as Foscarnet, block PRV DNA replication, physiological properties (resting potential, action potential shape) of cultured sensory neurons or in tissue explants are not altered [54],[55]. Blocking DNA replication prevents or at least significantly inhibits PRV late gene expression, which includes fusion proteins gB, gH, and gL. These findings are consistent with our finding that the PRV fusion machinery induces electrophysiological changes.

### A model for induced elevated AP firing rates and spikelet-like events *in vitro*

Our experiments directly demonstrate the development of electrical coupling between infected neurons, while results obtained with a gB null virus emphasize the importance of fusion in inducing increased firing. But how does electrical coupling and fusion produce an increased firing rate? We suggest that AP initiation in electrically isolated axonal compartments is crucial. Three lines of evidence support this assertion. First, hyperpolarization of cell bodies by injected current did not change AP firing rate, demonstrating that AP rate is not determined by successive depolarizations of the cell body membrane potential to AP threshold. Second, very strong hyperpolarization reduces AP amplitude, revealing a reduced AP originating in electrically distant compartments, which must be axons as these cells have no dendrites in culture [56]. Third, the shape of action potentials recorded in the cell bodies changes from a slow ramp to threshold to a sharp inflection (Figure 4B), a characteristic signature of a APs back-propagated from an axon to its cell body [31],[32],[56].

An important question is what induces APs in axons? More work is required to provide a definitive answer to this question but we note that the input resistance of an axon below threshold is normally very high [57]. We suggest small fusion pores formed between neurons, particularly those formed between axons, allow spontaneous APs generated in cell bodies to spread directly into coupled neurons. As APs normally occur at a low rate in uninfected neurons, an AP in one neuron could transfer from its axon into another coupled axon cause a brief depolarizing current which would result in either a spikelet-like event, or if sufficiently coupled a full AP, in the second axon. This signal would then travel to the cell body of the second axon, resulting in either a full back-propagated AP or spikelet-like event. This signal could then spread to other axons projecting from that cell, possibly inducing APs in the axons of other connected cells. In this way, APs could spread from cell to cell in the network allowing axonally initiated events to propagate throughout the network without attenuation. As a result, the number of APs per second would increase in any individually recorded neuron and also would lead to nearly synchronous activation of APs across neurons. In support of this model, spikelets and back propagated APs similar to those observed here have been observed in hippocampal neurons electrically and dye coupled by axo-axonal gap junctions [31]. Direct cytoplasmic continuity and ionic current flow mediated by gap junctions between neurons have been implicated in serving to support ultra-fast oscillations and synchronize neuronal firing [27],[32],[58]. Virally induced fusion pores could serve the same function, allowing activity to spread through a network of infected neurons. Although this model provides a qualitative understanding of the increased AP firing rate, it does not consider the effects of reverberation and feedback loops generated in the connected network. A numerical simulation using realistic compartmental modeling, or theoretical analysis using methods developed for understanding feedback neural circuits [31],[58],[59] could be used to examine this issue.

### Delayed onset of elevated AP firing rates and spikelet-like events in PRV 152 infected neurons

The delayed development of increased firing rates after PRV 152 infection is of some interest because it suggests that fusion/coupling events are modulated in some way by other viral gene products or processes. We have preliminary evidence to suggest that at least two processes are involved: axonal sorting of viral proteins and virus particle formation.

In work presented in the supplemental information (Figure S5, Protocol S1), we first demonstrated that the delayed onset of firing phenotype exhibited by PRV 152 is recessive by co-infecting neurons with PRV 151 and PRV 614 (PRV Bartha with RFP in place of GFP, Figure S5B). At 14–16 hpi, when PRV 152 infected neurons are firing at background levels and PRV 151 infected neurons are firing at a rapid rate, co-infected neurons fire at an elevated rate. Therefore, the PRV 152 genome contains mutations that delay onset of firing and these can be complemented by PRV 151 infection.

We and others have mapped and characterized several mutations in PRV Bartha responsible for its attenuated phenotype [16],[17],[18],[19],[21],[60]. These mutations were likely candidates for firing rate modifying genes. We used three recombinant viruses that carry defined segments of PRV Becker and PRV Bartha DNA to provide a first approximation of the localization of these modulating mutations (PRV BaBe, PRV 158 and PRV 327; Figure S5A).

The PRV Bartha Us region harbors a 3.4kb deletion removing the coding sequences for viral membrane proteins gE, gI, and Us9, which are required for localization of PRV structural proteins to axons and axonal membranes. As a consequence, in PRV Bartha infections, viral membrane fusion proteins including gB, gH, and gL are not present in axons [61],[62],[63],[64]. Accordingly, fusion events will be limited only to cell body-cell body or cell body-axon sites. This initial limitation in the number of sites where fusion can occur may reduce the number of sites where APs fired in one cell can induce an axonal AP in a coupled neuron. Consequently, the onset of elevated firing is delayed. We tested this prediction by infecting neurons with PRV 158 (the gE/gI/Us9 deletion in PRV Bartha is replaced with PRV Becker DNA). We observed that the early firing phenotype was restored but firing frequency was lower (Figure S5B). Sorting of viral proteins into axons is required for early induction of AP firing.

Recently, we reported that repair of the Bartha UL21 locus with wild-type sequence increased efficiency of transneuronal spread both *in vitro* and *in vivo* [60]. We found that UL21 mutations in PRV Bartha confer defects that affect infectious particle production, causing a delay in spread of infection to presynaptic neurons and subsequent amplification of infection. Remarkably, when we looked at firing rates after infection by PRV 327 (a PRV Bartha strain with only the UL21 locus replaced with PRV Becker DNA), we observed that early firing was restored, but again at a reduced rate (Figure S5B). We conclude that increased transneuronal spread leads to early AP firing events.

In work not shown, we infected neurons with PRV 4325, a recombinant PRV Bartha strain that carries the PRV Becker UL21 locus and the Becker Us region. The onset of firing and the rate was indistinguishable from PRV 151 infections. We conclude that reduced transneuronal spread (UL21 mutations) and absence of axonal localization of viral proteins (gE/gI/Us9 deletion) in PRV Bartha or PRV 152 infections are the primary contributors to the delayed firing phenotype.

### *In vitro* versus *in vivo* infections and electrophysiology

In contrast to our observations in PNS neurons *in vitro*, studies using attenuated PRV Bartha derivatives as neuronal circuit tracers report that infected neurons in CNS tissue display electrophysiological phenotypes indistinguishable from uninfected neurons [24],[25],[65]. It is critical to determine whether the effects we describe *in vitro* occur *in vivo*, as these strains are now being used to study circuit function using both electrical techniques and virally expressed functional indicators [66],[67].

Because PRV 152 neurons are significantly delayed in exhibiting increased firing events, the discrepancy between *in vitro* and *in vitro* observations may simply reflect the time at which neurons were analyzed. In a multi-synaptic circuit, it is difficult to establish the precise time of infection. The properties of infected neurons reported *in vivo* may have been measured during the early stages of PRV infection, before extensive neuronal coupling and associated elevated rates of firing began.

In addition, differences in the architecture of the infected neuronal circuit must be considered. If viral induced fusion can occur at any site of close membrane apposition (between cell bodies, or at synapses) where viral fusion proteins are present, then the extent of electrical coupling, fusion and un-attenuated AP firing will be limited by the quantity of potential fusion sites and circuit interconnectivity. In our *in vitro* system, neurons form an extensive, highly inter-connected network, free of any other cells, providing ample opportunity for fusion and AP propagation. *In vivo*, neurons are organized into functional circuits with varying degrees of inter-connectivity and often are ensheathed by glia, satellite cells or thick extracellular matrices [68]. Depending on the neuron and circuit in question, the quantity of potential sites for fusion and inter-connectedness of the circuit may dictate the timing and extent of fusion and elevated AP firing rates.

Alternatively, fusion and electrical coupling of infected CNS neurons may be revealed as more subtle changes in physiology, such as the synaptic integrity between connected neurons. One study by Glatzer el al. [23] reported that neurons of the nucleus tractus solitarius infected by PRV showed normal electrophysiological properties, but evoked postsynaptic currents of infected neurons had a significantly different latency and reproducibility. More recently, Boldogkoi et al [66] characterized the properties of neurons labeled with a PRV mutant (virulent background, deleted for gE/gI) expressing a genetically encoded activity sensor and a timing virus expressing a dsRed protein that is delayed by about 10 hours in maturing to form the fluorophore. They reported that retinal ganglion cells maintained normal electrophysiological properties over the first 10 hours of infection; however, by 10 hpi, light evoked photo-responses were significantly impaired. They took advantage of the onset of dsRed fluorescence to indicate the limit of when measurements could be taken. This timing ability will be useful in determining when if and when physiologically relevant measurements can be recorded from infected neurons *in vivo*.

### Biological implications

The dramatic peripheral neuropathy exhibited by PRV infected animals may reflect elevated rates of AP firing and neuronal fusion in neurons that innervate the infected tissue. Virulent strains of PRV lead to violent scratching at the site and dermatome of inoculation [6],[69]. Correspondingly, virulent PRV inoculation in the anterior chamber of the eye induces infected SCG neurons to fire at synchronously at elevated rates [6],[9]. These synchronous AP firing events are strongly reminiscent of our *in vitro* findings.

While infection with virulent PRV Becker induces pruritus in the mouse flank model of infection, the attenuated strain PRV Bartha does not [69]. PRV Bartha infects the sensory dorsal root ganglia; however, it does not spread to the dorsal horn of the spinal cord and from there through sensory routes to the brain as do the virulent strains of PRV. Infection of these neurons in the sensory pathway may result in abnormal sensory input, leading to scratching behavior. Because PRV Bartha cannot spread to these areas, no stimulus is induced and scratching behavior does not ensure. PRV Bartha does, however, invade the CNS from the skin via retrograde infection of descending pathways [69]. In the mouse model, signs of ataxia appear when PRV Bartha reaches the cerebellum, a highly interconnected region responsible for coordination. In highly connected circuits, electrophysiological changes induced by viral mediated fusion may be responsible for some of the neurological symptoms exhibited by infected animals.

Neuronal fusion has not been reported in PRV infected PNS ganglia or brain regions. However, previous assays, including electron microscopy, immuno-labeling and confocal microscopy, have relied on visual methods and did not measure fusion directly [7],[68]. Fusion of neurons has been shown *in vitro* and *in vivo* after infection with other viruses. Neuron-microglia fusion has been shown *in vivo* and *in vitro* by a replication incompetent retrovirus [70], while measles virus infection leads to fusion synaptic membranes *in vitro* [71]. Recently, fusion between neurons and satellite cells was reported in human sensory ganglia infected with varicella-zoster virus, a close relative of PRV [72].

Given our *in vitro* observations, neuronal fusion and associated changes in activity must be considered in PRV infected PNS ganglia and CNS nuclei. Future work will be directed at determining the extent to which changes in neuronal activity and connectivity occur in these tissues and their relevance to neurological symptoms central to pathogenesis.

## Materials and Methods

### Ethics statement

All experimental protocols related to animal use were approved by the Institutional Animal Care and Use Committee of the Princeton University Research Board under protocol number 1691 and are in accordance with the regulations of the American Association for Accreditation of Laboratory Animal Care and those in the Animal Welfare Act (public law 99–198).

### Cell lines and virus strains

All strains were propagated on porcine kidney (PK15) cells at a low multiplicity of infection for 48 hours and then collected into the conditioned medium as described previously [61]. Wild type PRV Becker [73] and its derivative PRV 151 expressing GFP were described previously [74]. PRV Bartha [20] and its derivative PRV 152 expressing GFP [24] were described previously. PRV 233 is recombinant virus created by crossing PRV HF22, a virus deleted for the UL27 gene encoding gB, and PRV 151 [26],[38]. This virus propagated on a gB complementing PK15 cell line (LP64e3). Mock inoculum stocks were prepared as above, but without adding virus.

### Primary neuronal cultures

Embryonic rat superior cervical ganglia (SCG) were isolated from Sprague-Dawley rats (Hill-Top Labs Inc., Scottdale, PA) between embryonic day 16.5 and E17.5 and dissociated into individual neurons as described previously [75]. In more cases the equivalent of one ganglia was plated in the center (in 100 µl, ≈0.5cm) of a 35-mm plastic tissue culture dishes (BD Falcon, New Jersey) coated with 500 g/ml of poly-DL-ornithine (Sigma Aldrich) diluted in borate buffer and 10g/ml of natural mouse laminin (Invitrogen). Dissociated neurons were allowed to settle for 5 minutes prior to addition of 2 ml of medium. Neuron culture medium used was either serum-free medium as previously described [75] or Neurobasal (Invitrogen) supplemented with 100 ng/ml nerve growth factor, 1× B27 (Invitrogen), 2mM L-glutamine (J. T. Baker) and 100 ng/mL penicillin and streptomycin (P/S). On a few occasions, SCGs were stored for less than one week in Hibernate E minus Calcium (BrainBits, Illinois) before dissociation. In all cases, 1–2 days after plating, cultures were exposed to the antimitotic drug, cytosine-D-arabinofuranoside (1 µM, Sigma-Aldrich), for 1–2 days to kill contaminating epithelial or support cells. Cultures were maintained in a humidified incubator at 37°C with 5% CO_2_ at (Thermo-Scientific). Medium was replaced every 3–5 days.

### Viral infection of neuronal cultures

Infection of neurons was performed as described previously [75]. Cultures were inoculated with 10^6^ plaque forming units (in 600 µl over entire 35 mm dish) and incubated for 1 hour prior to replacement with neuronal medium. Mock-infection consisted of either addition of mock inoculum described above or DMEM supplemented with 2% FBS and 1% P/S.

### Electrophysiological recordings

Whole-cell patch clamp recordings were performed at 37°C on neurons grown in culture for 10–20 days. Before recording, neuronal medium was replaced with warmed physiological extracellular solution, (mM) 146 NaCl, 4.7 KCl, 1.6 NaHCO_3_, 0.13 NaH_2_PO_4_, 10 HEPES, 7.8 glucose, 2.5 CaCl_2_ and 0.6 MgSO_4_ (OSM 320) pH 7.2. A section of culture dish side-wall was removed by melting a small section to allow unobstructed pipette access to the culture dish. Extracellular solution was maintained at 37°C (±2°C) by placing the culture dish on a heated copper plate with supporting walls and monitored during recordings (HH-25TC Thermometer, Omega). Patch pipettes pulled (P-2000, Sutter Instruments) from borosilicate glass (30-31-1, FHC, Bowdoinham, ME) to a resistance of 4–7MΩ were filled (mM) with intracellular solution, 140 K-gluconate, 10 HEPES, 2 MgCl_2_, 3 ATPNa_2_ and 0.3 GTPNa (OSM 275) pH 7.3 (Sigma-Aldrich). Prior to and during recordings neurons were visualized with a 40× water immersion objective (Olympus LUMPIPlanFI/IR 40× 0.80) using an Olympus BX51WI microscope. Whole-cell patches were obtained from soma of individual neurons in clusters of 2 to 8 after 1–4 GΩ seals were formed. Stable whole cell recordings were observed for 1–5 min prior to recording of spontaneous activity and resting membrane potentials with a Dagan N = 0.1 or N = 0.01 headstage and BVC700 amplifier for 2–5 minutes in current clamp mode with no holding current. Next, a series of 1s current injections (−0.15nA to 0.15nA) were delivered every 10 seconds under current clamp from resting voltage. Capacitance and series (5–40MΩs) resistance were compensated. In several occasions uncompensated series resistance was subtracted off-line prior to analysis. Recordings from cells with >10 mV baseline changes through the duration of spontaneous activity were rejected. Current injection data from neurons showing this instability were also rejected. Dual recordings were obtained using the same procedure as above with a second electrode, headstage and amplifier. Following recordings, cells were briefly viewed for GFP fluorescence to confirm infection (Olympus FITC/GFP filter cube).

### Electrophysiology data acquisition and analysis

Data were obtained and digitized at 20kHz using an Axon Digidata 1233a and Clampex 9 or 1440a and Clampex 10 software (Axon/Molecular Devices). Junction potential was calculated as −17 mV using Clampex software's junction potential assistant and subtracted from all raw voltage data prior to analysis. All data were analyzed offline using custom MATLAB software.

Resting voltage was determined as follows: for silent neurons (0 Hz) resting voltages were determined by averaging voltage across the entire recording, for neurons with events occurring between >0 and <1 Hz the mean voltage across the entire measurement except for times when the resting membrane potential was greater than ±SEM (during action potentials/AHP), and for neurons showing events >1Hz the mean voltage during of periods of steady voltage, recalculated and confirmed by manual inspection of >3 1 s intervals. Action potential firing rate was determined from whole-cell recordings of 120 seconds, defined as the number of large voltage deflections crossing a threshold (>50 mV from baseline) divided by total time. AP amplitude was defined at the peak voltage following threshold crossing minus resting voltage, AHP was defined as the difference between resting voltage (baseline) and the voltage 11 ms following AP peak. AP height was defined as the difference between AP peak and voltage 11 ms following the peak.

Spikelet-like events were identified by a threshold imposed on the first derivative of the smoothed voltage trace (4–20V/s). Identified events were then manually confirmed by visual inspection based on determination of maximum rise rate and time to half decay from the peak. Rates of either event (APs and spikelet-like events) were determined as the number of events divided by total recording time analyzed. Total event rate was defined as the sum of these three classes of events. Total event rates during current injections were determined as above and reported as the ratio of event rate to the maximum event rate for each cell (up to 0.15nA). Steady state voltage during injections with <3 events was defined as the mean voltage during the last 200 ms. For those injections eliciting >3 events, steady state voltage was determined as the mean voltage during the last 200 ms excluding events.

Input resistance was calculated using the linear fit of steady state resting voltages recorded during injections to current injections. AP threshold was calculated by subtracting the steady state voltage during the lowest positive current injection eliciting at least one AP. Slopes were taken over 10 mV bins to account for starting resting voltage differences between treatments. DC coupling ratios were determined by current injections into one cell body during dual recordings. Values reported are the mean voltage deflection of the second cell divided by the mean voltage deflection of the directly injected cell. All data reported as ±SEM. Student t-tests or unpaired two-tailed t-tests were used to determine significance, *P<0.05.

### Fluorescent dyes and image acquisition

Whole-cell patch clamp recordings were performed as above with 0.5 ug/ml neutral Texas-red Dextran conjugates at 3000, 10000 or 40000 MW (Invitrogen) or 0.05% Lucifer yellow (LY), MW 457 (Invitrogen). In LY experiments, dye was allowed to diffuse from the patch pipette into the neuron for 10–15 minutes. The pipette was then removed and the extracellular medium was replaced. After one hour, fluorescence images were taken at 40× of the filled cell body and its processes, at multiple focal planes. Resulting images were assessed for additional labeled cell bodies and contiguous labeled processes were counted. In large MW dye experiments, dye was allowed to diffuse from the patch pipette and images were recorded immediately, in most cases prior to pipette removal. Images were taken at multiple planes and assessed for additional labeled cell bodies. All images were obtained using an Olympus Bx51WI microscope by a CoolSnapcf CCD camera (Photometrics) and RS Image (Roper Scientific). Fluorescence images were acquired using automatic exposure with an intensity target of 3000, limit 1s, gain 1. Images of GFP expressed from the viral genome, Texas-red or Lucifer Yellow from patch pipette, and bright-field images using Olympus HQ:FITC or HC Red filter cubes. Fluorescence images were pseudo-colored as appropriate and enhanced for contrast at 0.5% saturated pixel intensity using ImageJ (NIH). Cell bodies were considered to have dye transferred from the filled cell body if their mean intensity was three times the standard deviation of background. Resulting images were screened onto bright field images obtained at the same focal plane using Adobe Photoshop 10.0.

### Large-scale neuronal fusion assay

Percentage of fused neurons was determined by a combination of the angle measured between cell bodies and, for heavily fused cultures, by the area of large rounded neuronal syncytial. The angle between the outer edge of adjacent neurons was first determined in pairs of neurons that were tested for large MW dye coupling using ImageJ. These measurements resulted in clear separation between fused (>125 degrees) and unfused neurons (∼50–∼115 degrees, Figure S4A,B). This separation allowed us to determine whether unfilled cell bodies from randomly chosen 10× fields of view were fused (Olympus UMPPlanFI 10× 0.30). For large structures (syncytia) observed late (40 & 78 hpi, Figure 8A, Figure S4C) no angle could be measured. To determine the number of fused neurons in this case, a standard curve was constructed by measuring two dimensional area and fitting to a curve of known area to nuclei. Hoechst 33342 (Invitrogen) was added at 1 ug/ml in neuronal media for 20 min, washed twice with extracellular solution prior bright field and fluorescence imaging. Images were collected over several focal planes to count the number of nuclei and the two dimensional area of multinucleated syncytia was measured using ImageJ. The resulting standard curve was created using MATLAB (Figure S4D). Single neurons not in clusters, as defined as having an area less than the maximum area of a cell body with a single nucleas (<300 µm^2^), were excluded from calculation the percentage of cell bodies fused. The standard curve allowed us to determination of minimum number of nuclei (cells fused) in area measurements from random unstained fields of syncytial clusters of neurons. The total number of cell bodies counted as fused by either method was divided by the total number assayed.

## Supporting Information

Protocol S1 (0.07 MB PDF)

Figure S1 AP and spikelet-like event responses of PRV infected neuron to cell body current injections late in PRV 151 infection. (A–D) Relationship between injected current and AP rate during current injections. The rate of APs for each current injection (between I = −0.15 and 0.14nA) was divided by the maximum event rate for that cell over all injections. Ratios were averaged and plotted against injected current. Note increasingly unchanged ratios in PRV 151 infected neurons as a function of time after infection. (E–F) Ratios of AP rate/max AP rate for individual neurons fall into several categories late in infection, leading to large error bars in A–D. (E) At 14–16 hpi, 10/15 PRV 151 infected neurons showed very little change in ratio over the range of hyperpolarizing and depolarizing currents, example neuron marked with circled #1. 5/10 showed a relationship similar to that observed at 8–10 hpi, example neuron marked with #2. F) At 24–26 hpi, PRV 151 infected neurons showed AP firing rates largely independent of current injection (5/7), examples labeled as in E. (G–J) Relationship between injected current and total (AP and spikelet-like) event rate during current injections. The rate of total APs and spikelet-like events for each current injection (between I = −0.15 and 0.14nA) was divided by the maximum event rate for that cell over all injections. Ratios were averaged and plotted against injected current. As above, note increasingly unchanged ratios in infected neurons as a function of time after infection. (B–E) Mock n = 24, 4–8 hpi PRV 151 n = 9, 8–10 hpi n = 7, 14–16 hpi n = 15, 24–26 hpi n = 7. Plots were generated using data from Figure 3.
(0.55 MB TIF)

Figure S2 Infected neurons that share large MW dyes are strongly electrically coupled. Near complete electrical coupling is seen when large MW dye is able to transfer to an adjacent cell body. Voltage response of a second neuron was divided by the first neurons response to direct current injection. Examples shown are from PRV 151 infected neurons at 18–20 hpi. (A) A pair of infected neurons with no large MW transfer shows a low level of electrical coupling. (B) A pair of infected neurons with large MW dye transfer shows a high level of electrical coupling.
(0.24 MB TIF)

Figure S3 Criteria for cell body-cell body fusion. (A) Histogram of measured angles between adjacent cell bodies. Angles were measured from cell pairs in which large MW did or did not transfer, at 24 hpi with either PRV151 or PRV 152. Neuron pairs with angles less than 125° were determined not fused and pairs with larger angles were determined fused. Mock-infected neurons had a similar distribution of angles. (B) Examples of measured angles, one each of fused and not fused used in calculation of % of cell bodies fused. (C) Examples of extensive fusion of clustered neurons at 40 hpi for both PRV 151 and PRV 152 infected neurons. Blue - Hoechst, (40×). Example of two-dimensional area measured, cell indicated by an asterisk in D. To estimate the number of fused cells in a syncytia, we used the minimum number of nuclei observed for measured area to avoid over counting. (D) Standard curve constructed from two-dimensional area calculated and the number of nuclei counted. Cells with areas larger than 300 µm^2^, larger than maximum size of a single neuron, indicated by the dotted line, were counted as fused. Those with smaller areas adjacent to other cell bodies, were counted as not fused and those with smaller areas not adjacent to other cell bodies were not included in percentages presented in Figure 8B).
(1.29 MB TIF)

Figure S4 AP rate responses of PRV infected neuron to cell body current injections late in PRV 152 infection. A–D) Relationship between injected current and AP rate during current injections. The rate of APs for each current injection (between I = −0.15 and 0.14nA) was divided by the maximum event rate for that cell over all injections. Ratios were averaged and plotted against injected current. Note unchanged ratios in PRV 152 infected neurons are delayed until 24–26 hpi. E–F) Ratios of AP rate/max AP rate for individual neurons fall into several categories late in infection, leading to large error bars in A–D. E) At 14–16 hpi, 13/19 PRV 152 infected neurons responded to positive current injections with increased AP firing (#3), while 6/19 showed little change in rate over the range of voltages (#4). F) By 24–26 hpi, 5/8 PRV 152 infected neurons did not respond to current injections by firing increased APs. 4/8 of these had very high levels of spikelet-like events and did not fire APs during any injection and showed no change in spikelet-rate across injections (J, Figure 8B). 3/8 PRV 152 infected neurons did show a slight increase in AP rate in response to more positive current injections (#3). G–J) Relationship between injected current and total (AP and spikelet-like) event rate during current injections. The rate of total APs and spikelet-like events for each current injection (between I = −0.15 and 0.14nA) was divided by the maximum event rate for that cell over all injections. Ratios were averaged and plotted against injected current. Note increasingly unchanged ratios in infected neurons as a function of time after infection. The total event range was not changed by current injection in all categories of neurons infected with either strain at 24–26 hpi (B–J) PRV 152 4–8 hpi n = 7, 8–10 hpi n = 7, 24–26 hpi n = 8. Plots were generated using data from Figures 3 and 8.
(0.63 MB TIF)

Figure S5 Multiple alleles of PRV Becker contribute to early onset of elevated AP firing rates. (A) Genotype maps of PRV Becker and PRV Bartha recombinants assayed in B. (B) Mean AP firing rates of infected neurons at 14–16 hpi. PRV 151 + PRV 614 n = 6, PRV 158 n = 8, PRV BaBe n = 10, PRV 327 n = 7.
(0.50 MB TIF)
