# Supplementary material for: Pseudorabies Virus Infection Alters Neuronal Activity and Connectivity In Vitro
Source: PLoS Pathog. 2009 Oct 30;5(10):e1000640. doi: 10.1371/journal.ppat.1000640 (PMC2763221; doi:10.1371/journal.ppat.1000640)
Supplement: Protocol S1 — (0.07 MB PDF) [file ppat.1000640.s001.pdf]

## Supplemental Methods

**Virus strains.** PRV 614 is PRV Bartha with gG replaced with RFP [1], PRV 158 PRV 158 contains the unique long (UL) region of Bartha and the unique short (US) region of Becker [2], PRV BaBe is Becker containing the US deletion of Bartha [3], PRV 43/25 aB4 is Bartha with the wild-type PRV Kaplan sequence restoring BamHI fragment 4 and the US region [4], PRV 327 is PRV 43/25 aB4 with the US deletion of Bartha reintroduced, and gG replaced with GFP [3,5].

## Supplementary References

1. Banfield BW, Kaufman JD, Randall JA, Pickard GE (2003) Development of pseudorabies virus strains expressing red fluorescent proteins: new tools for multisynaptic labeling applications. J Virol 77: 10106-10112.
2. Lyman MG, Demmin GL, Banfield BW (2003) The attenuated pseudorabies virus strain Bartha fails to package the tegument proteins Us3 and VP22. J Virol 77: 1403-1414.
3. Card JP, Whealy ME, Robbins AK, Enquist LW (1992) Pseudorabies virus envelope glycoprotein gI influences both neurotropism and virulence during infection of the rat visual system. J Virol 66: 3032-3041.
4. Lomniczi B, Watanabe S, Ben-Porat T, Kaplan AS (1984) Genetic basis of the neurovirulence of pseudorabies virus. J Virol 52: 198-205.
5. Curanovic D, Lyman MG, Bou-Abboud C, Card JP, Enquist LW (2009) Repair of the UL21 locus in pseudorabies virus Bartha enhances the kinetics of retrograde, transneuronal infection in vitro and in vivo. J Virol 83: 1173-1183.
