# Supplementary figures and images for: Pseudorabies Virus Infection Alters Neuronal Activity and Connectivity In Vitro
Source: PLoS Pathog. 2009 Oct 30;5(10):e1000640. doi: 10.1371/journal.ppat.1000640 (PMC2763221; doi:10.1371/journal.ppat.1000640)

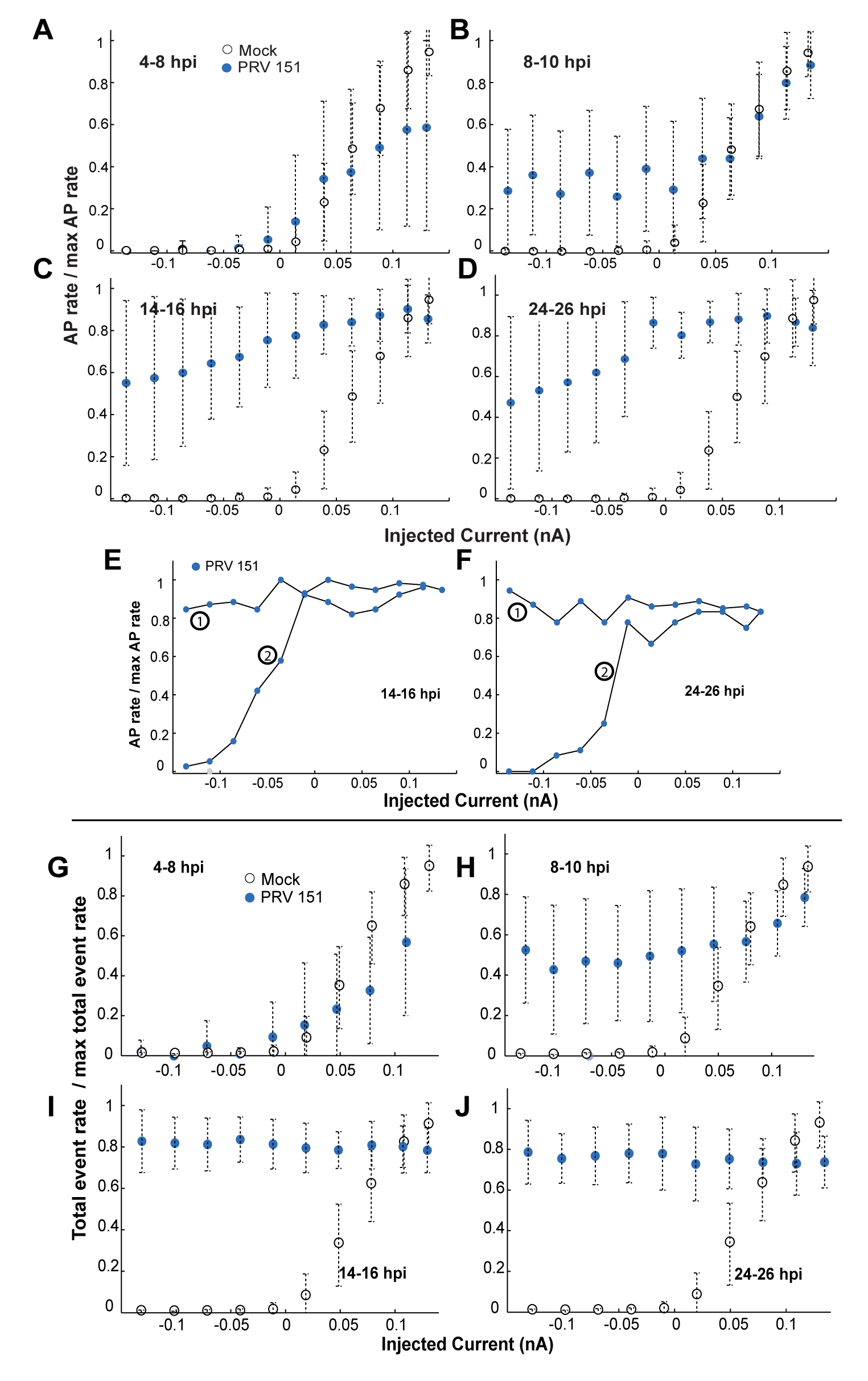

Supplement: Figure S1 — AP and spikelet-like event responses of PRV infected neuron to cell body current injections late in PRV 151 infection. (A–D) Relationship between injected current and AP rate during current injections. The rate of APs for each current injection (between I = −0.15 and 0.14nA) was divided by the maximum event rate for that cell over all injections. Ratios were averaged and plotted against injected current. Note increasingly unchanged ratios in PRV 151 infected neurons as a function of time after infection. (E–F) Ratios of AP rate/max AP rate for individual neurons fall into several categories late in infection, leading to large error bars in A–D. (E) At 14–16 hpi, 10/15 PRV 151 infected neurons showed very little change in ratio over the range of hyperpolarizing and depolarizing currents, example neuron marked with circled #1. 5/10 showed a relationship similar to that observed at 8–10 hpi, example neuron marked with #2. F) At 24–26 hpi, PRV 151 infected neurons showed AP firing rates largely independent of current injection (5/7), examples labeled as in E. (G–J) Relationship between injected current and total (AP and spikelet-like) event rate during current injections. The rate of total APs and spikelet-like events for each current injection (between I = −0.15 and 0.14nA) was divided by the maximum event rate for that cell over all injections. Ratios were averaged and plotted against injected current. As above, note increasingly unchanged ratios in infected neurons as a function of time after infection. (B–E) Mock n = 24, 4–8 hpi PRV 151 n = 9, 8–10 hpi n = 7, 14–16 hpi n = 15, 24–26 hpi n = 7. Plots were generated using data from Figure 3. (0.55 MB TIF) [file ppat.1000640.s002.tif]

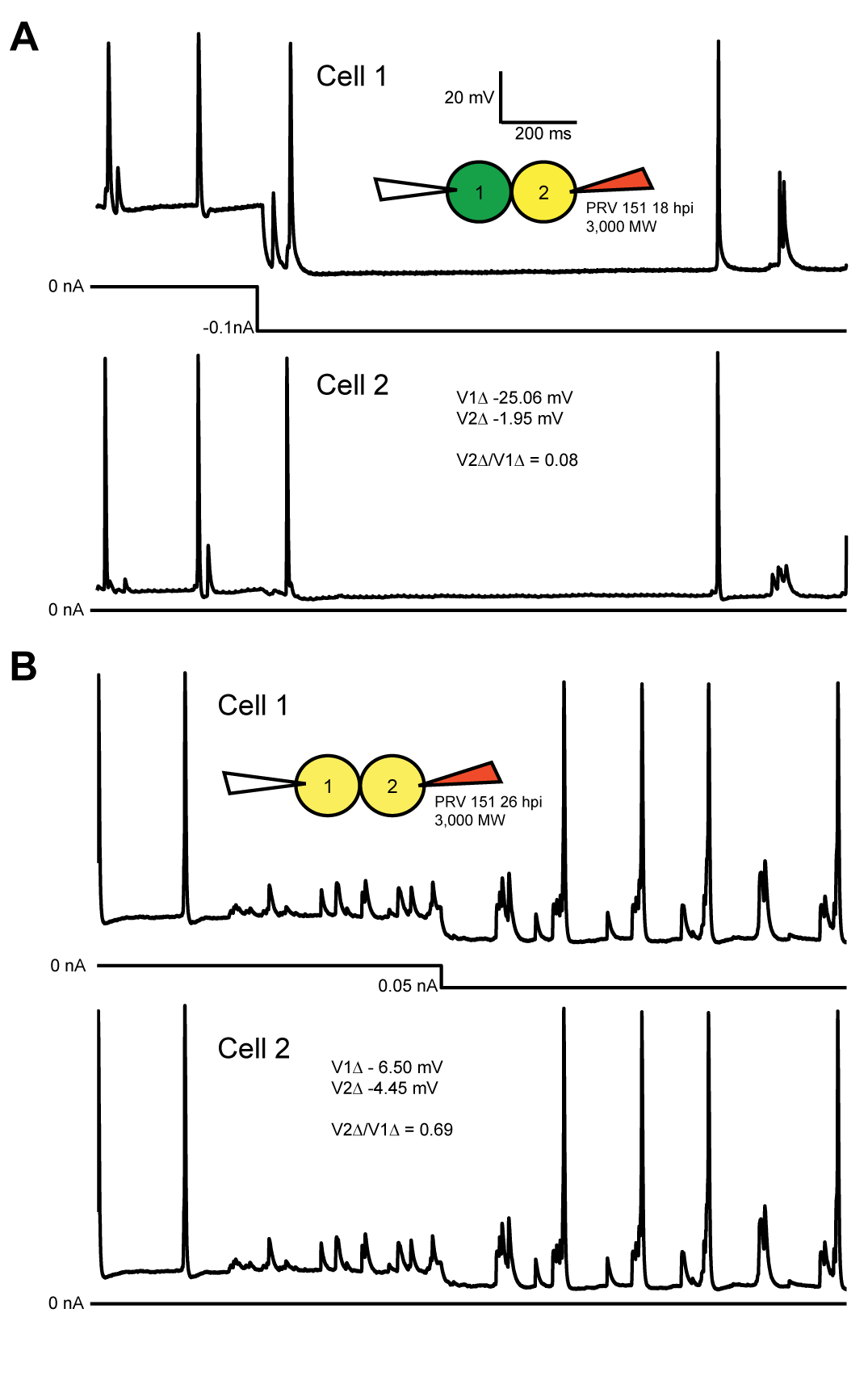

Supplement: Figure S2 — Infected neurons that share large MW dyes are strongly electrically coupled. Near complete electrical coupling is seen when large MW dye is able to transfer to an adjacent cell body. Voltage response of a second neuron was divided by the first neurons response to direct current injection. Examples shown are from PRV 151 infected neurons at 18–20 hpi. (A) A pair of infected neurons with no large MW transfer shows a low level of electrical coupling. (B) A pair of infected neurons with large MW dye transfer shows a high level of electrical coupling. (0.24 MB TIF) [file ppat.1000640.s003.tif]

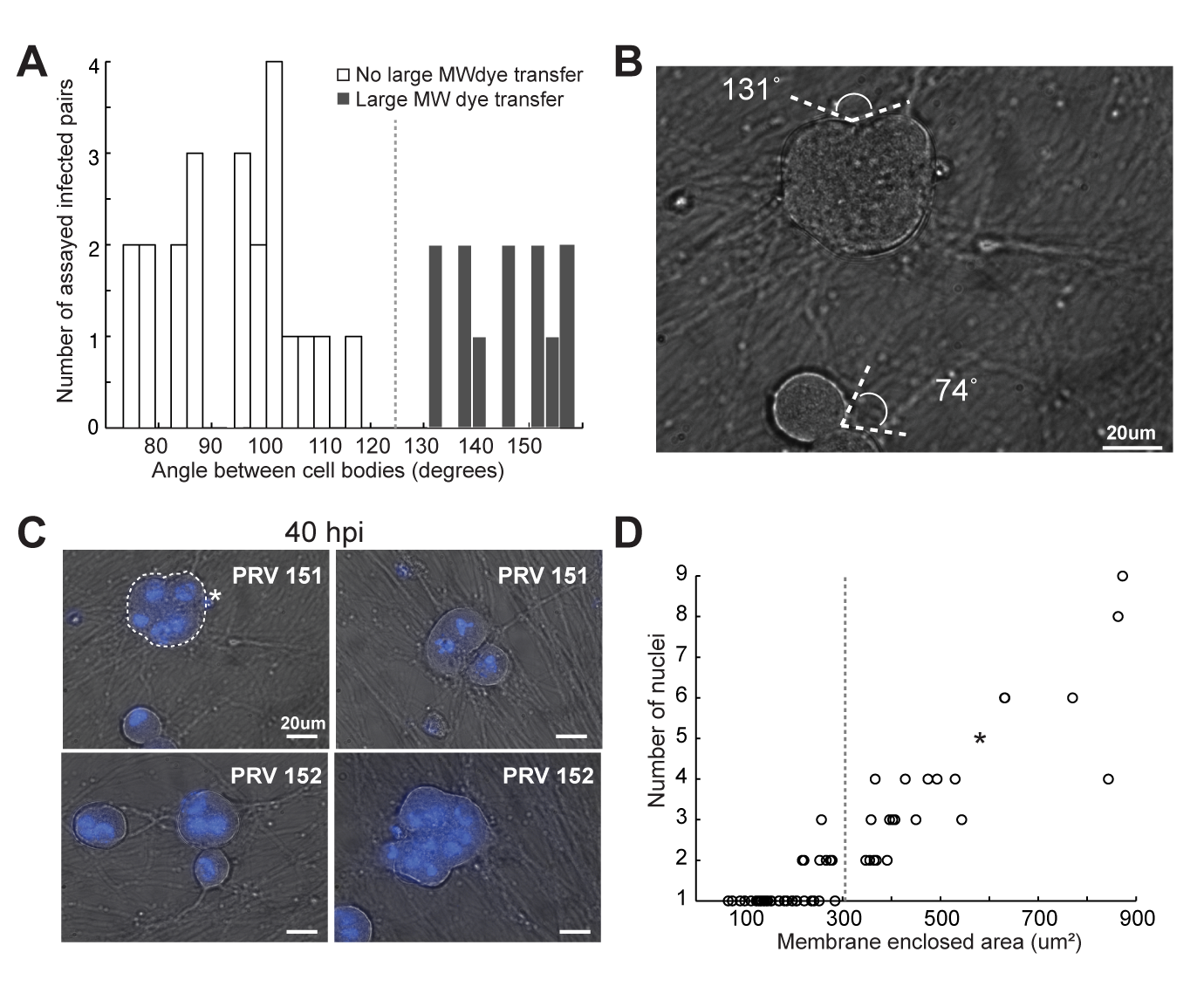

Supplement: Figure S3 — Criteria for cell body-cell body fusion. (A) Histogram of measured angles between adjacent cell bodies. Angles were measured from cell pairs in which large MW did or did not transfer, at 24 hpi with either PRV151 or PRV 152. Neuron pairs with angles less than 125° were determined not fused and pairs with larger angles were determined fused. Mock-infected neurons had a similar distribution of angles. (B) Examples of measured angles, one each of fused and not fused used in calculation of % of cell bodies fused. (C) Examples of extensive fusion of clustered neurons at 40 hpi for both PRV 151 and PRV 152 infected neurons. Blue - Hoechst, (40×). Example of two-dimensional area measured, cell indicated by an asterisk in D. To estimate the number of fused cells in a syncytia, we used the minimum number of nuclei observed for measured area to avoid over counting. (D) Standard curve constructed from two-dimensional area calculated and the number of nuclei counted. Cells with areas larger than 300 µm2, larger than maximum size of a single neuron, indicated by the dotted line, were counted as fused. Those with smaller areas adjacent to other cell bodies, were counted as not fused and those with smaller areas not adjacent to other cell bodies were not included in percentages presented in Figure 8B). (1.29 MB TIF) [file ppat.1000640.s004.tif]

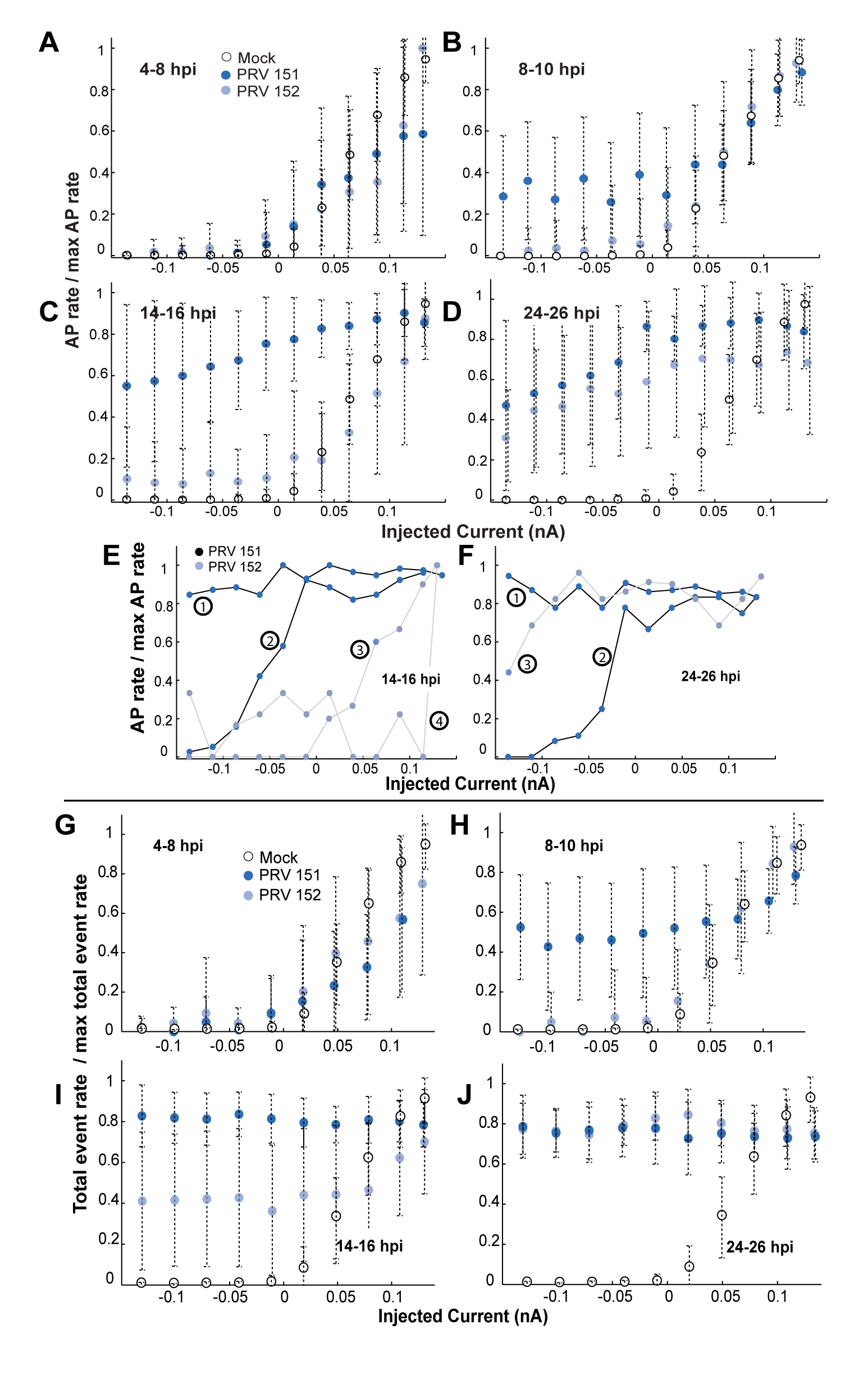

Supplement: Figure S4 — AP rate responses of PRV infected neuron to cell body current injections late in PRV 152 infection. A–D) Relationship between injected current and AP rate during current injections. The rate of APs for each current injection (between I = −0.15 and 0.14nA) was divided by the maximum event rate for that cell over all injections. Ratios were averaged and plotted against injected current. Note unchanged ratios in PRV 152 infected neurons are delayed until 24–26 hpi. E–F) Ratios of AP rate/max AP rate for individual neurons fall into several categories late in infection, leading to large error bars in A–D. E) At 14–16 hpi, 13/19 PRV 152 infected neurons responded to positive current injections with increased AP firing (#3), while 6/19 showed little change in rate over the range of voltages (#4). F) By 24–26 hpi, 5/8 PRV 152 infected neurons did not respond to current injections by firing increased APs. 4/8 of these had very high levels of spikelet-like events and did not fire APs during any injection and showed no change in spikelet-rate across injections (J, Figure 8B). 3/8 PRV 152 infected neurons did show a slight increase in AP rate in response to more positive current injections (#3). G–J) Relationship between injected current and total (AP and spikelet-like) event rate during current injections. The rate of total APs and spikelet-like events for each current injection (between I = −0.15 and 0.14nA) was divided by the maximum event rate for that cell over all injections. Ratios were averaged and plotted against injected current. Note increasingly unchanged ratios in infected neurons as a function of time after infection. The total event range was not changed by current injection in all categories of neurons infected with either strain at 24–26 hpi (B–J) PRV 152 4–8 hpi n = 7, 8–10 hpi n = 7, 24–26 hpi n = 8. Plots were generated using data from Figures 3 and 8. (0.63 MB TIF) [file ppat.1000640.s005.tif]

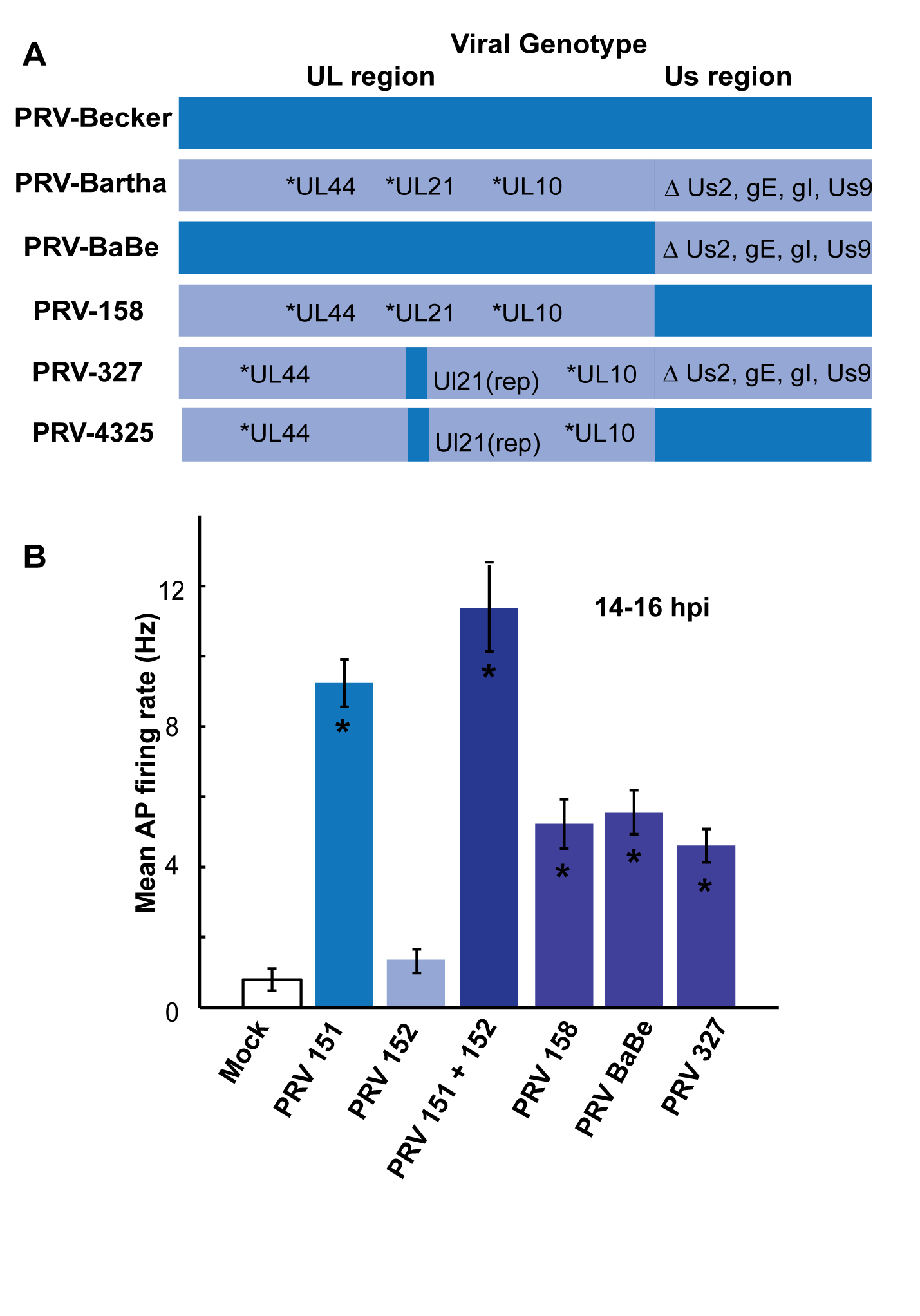

Supplement: Figure S5 — Multiple alleles of PRV Becker contribute to early onset of elevated AP firing rates. (A) Genotype maps of PRV Becker and PRV Bartha recombinants assayed in B. (B) Mean AP firing rates of infected neurons at 14–16 hpi. PRV 151 + PRV 614 n = 6, PRV 158 n = 8, PRV BaBe n = 10, PRV 327 n = 7. (0.50 MB TIF) [file ppat.1000640.s006.tif]
